# A Family of Toxoplasma gondii Genes Related to GRA12 Regulate Cyst Burdens and Cyst Reactivation

**DOI:** 10.1128/mSphere.00182-21

**Published:** 2021-04-21

**Authors:** Rebekah B. Guevara, Barbara A. Fox, David J. Bzik

**Affiliations:** a Department of Microbiology and Immunology, The Geisel School of Medicine at Dartmouth, Lebanon, New Hampshire, USA; University of Georgia

**Keywords:** *Toxoplasma gondii*, dense granules, intravacuolar network, bradyzoite differentiation, cysts, chronic infection, cyst wall, cyst matrix, cyst development, cyst persistence, cyst reactivation, GRA12, dense granule, virulence

## Abstract

Toxoplasma gondii causes a chronic infection that renders the immunocompromised human host susceptible to toxoplasmic encephalitis triggered by cyst reactivation in the central nervous system. The dense granule protein GRA12 is a major parasite virulence factor required for parasite survival during acute infection. Here, we characterized the role of four GRA12-related genes in acute and chronic stages of infection. While GRA12A, GRA12B, and GRA12D were highly expressed in asexual stage tachyzoites and bradyzoites, expression of GRA12C appeared to be restricted to the sexual stages. In contrast to deletion of GRA12 (Δ*gra12*), no major defects in acute virulence were observed in Δ*gra12A*, Δ*gra12B*, or Δg*ra12D* parasites, though Δ*gra12B* parasites exhibited an increased tachyzoite replication rate. Bradyzoites secreted GRA12A, GRA12B, and GRA12D and incorporated these molecules into the developing cyst wall, as well as the cyst matrix in distinct patterns. Similar to GRA12, GRA12A, GRA12B, and GRA12D colocalized with the dense granules in extracellular tachyzoites, with GRA2 and the intravacuolar network in the tachyzoite stage parasitophorous vacuole and with GRA2 in the cyst matrix and cyst wall. Chronic stage cyst burdens were decreased in mice infected with Δ*gra12A* parasites and were increased in mice infected with Δ*gra12B* parasites. However, Δ*gra12B* cysts were not efficiently maintained *in vivo*. Δ*gra12A*, Δ*gra12B*, and Δ*gra12D in vitro* cysts displayed a reduced reactivation efficiency, and reactivation of Δ*gra12A* cysts was delayed. Collectively, our results suggest that a family of genes related to GRA12 play significant roles in the formation, maintenance, and reactivation of chronic stage cysts.

**IMPORTANCE** If host immunity weakens, Toxoplasma gondii cysts recrudesce in the central nervous system and cause a severe toxoplasmic encephalitis. Current therapies target acute stage infection but do not eliminate chronic cysts. Parasite molecules that mediate the development and persistence of chronic infection are poorly characterized. Dense granule (GRA) proteins such as GRA12 are key virulence factors during acute infection. Here, we investigated four GRA12-related genes. GRA12-related genes were not major virulence factors during acute infection. Instead, GRA12-related proteins localized at the cyst wall and cyst matrix and played significant roles in cyst development, persistence, and reactivation during chronic infection. Similar to GRA12, the GRA12-related proteins selectively associated with the intravacuolar network of membranes inside the vacuole. Collectively, our results support the hypothesis that GRA12 proteins associated with the intravacuolar membrane system support parasite virulence during acute infection and cyst development, persistence, and reactivation during chronic infection.

## INTRODUCTION

Toxoplasma gondii is an obligate intracellular protozoan pathogen that chronically infects warm-blooded mammalian and avian hosts, including one-third of the global human population (1). Chronic infection is efficiently established and marked by the formation of thick-walled tissue cysts, which mediate parasite transmission to new hosts. *Toxoplasma* infections are acquired through oral ingestion of tissue cysts found in undercooked meat, through consumption of oocysts from cat feces in contaminated water or on unwashed fruits and vegetables, or through maternal to fetal transmission (2). Primary infection during pregnancy causes severe congenital defects in the newborn (3, 4). Humans are typically asymptomatic, since infection is controlled by the immune system. However, if infection occurs in the immune-privileged eye, recurrent ocular toxoplasmosis leads to visual impairment (5). Chronically infected AIDS, cancer, and transplant patients who experience weakened immunity become susceptible to cyst reactivation in the brain, which can develop into a fatal reactivated infection causing toxoplasmic encephalitis (6, 7). Therapeutic interventions to target the chronic cyst stage are not yet available.

*Toxoplasma* efficiently invades a vast variety of nucleated host cells using an arsenal of secreted effectors stored in secretory organelles (8). These secreted effector proteins are released from secretory organelles called rhoptries, micronemes, and dense granules (the organelle containing GRA proteins) in a coordinated fashion to mediate the parasite’s attachment and invasion into host cells, as well as the establishment of the intracellular parasitophorous vacuole (PV) that supports parasite survival and replication inside the host cell (9–11). The dense granules are massively secreted into the PV space shortly after the formation of the PV (12), releasing a large repertoire of GRA proteins as well as some initial membranous material, which together with lipids recruited from the host cell form an extensive intravacuolar network (IVN) of membranes that forms connections inside the PV between parasites and the limiting PV membrane (PVM) (13–15). While all IVN-associated GRAs have the capability to associate with and disassociate from the IVN membranes in the PV space (12), certain GRAs such as GRA2, GRA4, GRA6, GRA9, and GRA12 strongly associate with the IVN membranes typically through transmembrane interactions, whereas other GRAs such as GRA1 weakly associate with the IVN membranes through peripheral membrane interactions. Many other GRAs are secreted past the IVN and PV space and associate with the PVM at the host-PV interface (12) or passage through the PVM (translocate) into the host cytosol (GRA18). A number of GRAs that translocate past the PVM, including GRA16, GRA24, and GRA28, target to and accumulate in the host cell nucleus (12, 16).

GRAs shape the functions of the PV and the host cell environment to promote PV and parasite survival, nutrient uptake, and parasite replication (15, 17–20). For example, GRA17 and GRA23 facilitate the movement of small molecules across the PVM (21, 22), and F-actin filaments, contained in IVN membrane tubules organized by GRA2 and GRA6 (14), are involved in moving vesicles between tachyzoites in the PV (23, 24). Several IVN-associated GRA genes, including GRA1, GRA2, GRA6, and GRA12, were proposed to contribute to *Toxoplasma* pathogenesis when transcriptomic data of *Toxoplasma* were analyzed using a computational gene regulatory network approach (25). Indeed, GRA12 (TgME49_288650) does contribute to *Toxoplasma* pathogenesis, as GRA12 was recently identified as a major virulence factor that resists host interferon gamma (IFN-γ) to promote parasite survival during acute infection (26). Furthermore, a virulence factor screen has confirmed the key role of GRA12 in mediating parasite survival during *in vivo* infection (27). GRA12 displays little homology to proteins that are not expressed by Coccidian species, which suggests a specificity to the functional role(s) of GRA12 in Coccidians.

GRA12 was first identified as an IVN-associated GRA based on its pattern of secretion, colocalization, and biochemical fractionation with IVN membranes as a transmembrane and PV soluble protein (28). Previously, three additional *Toxoplasma* GRA12-related sequences (TgME49_220890, TgME49_275860, and TgME49_275850) were reported to display significant homology with GRA12 and were proposed as probable GRA12 gene paralogs (28). Herein, we identify a fourth GRA12-related gene (TgME49_308970). Recently reported GRA17-BirA* and GRA25-BirA* pulldowns identified TgME49_275860 as a novel PV resident protein in tachyzoites (16). In addition, GRA1-BirA* pulldowns identified TgME49_275860, while the GRA1-APEX fusion identified TgME49_220890 and TgME49_308970 as novel PV proteins (29).

In addition to their key roles in acute infection, the role of GRAs in the chronic stage of infection is evident by their presence in tissue cysts at the cyst membrane, cyst wall, and within the cyst matrix (30–33). Mature cysts possess a thick cyst wall structure, which is organized into two distinct filamentous layers, a more densely compacted outer layer beneath a limiting cyst membrane and a less densely compacted inner layer that faces the cyst matrix (34). The major cyst wall glycoprotein, CST1, contains a mucin domain that is heavily decorated with *N*-acetylgalactosamine moieties that are recognized by Dolichos biflorus agglutinin (DBA) stain (35–37). The cyst matrix contains soluble components, filamentous materials, membranous tubules, and vesicles that occupy the space between bradyzoites and the cyst wall (34). GRA2 has an important role in organizing the cyst matrix (30). Deletion of PVM- or IVN-associated GRA proteins significantly reduces cyst burdens, suggesting that membrane-associated GRA proteins provide important functions for cyst development and persistence (26, 38). Furthermore, deletion of GRA12 or GRA50/CST2 completely abolished cyst burdens (26, 32). However, the specific role of GRA12 during a chronic infection is challenging to elucidate because GRA12 is required for PV resistance to host IFN-γ during the acute stage of infection (26).

In this study, we investigated a family of four GRA12-related genes: TgME49_220890, TgME49_275860, TgME49_275850, and TgME49_308970. Our findings show that TgME49_275850 is not expressed in the tachyzoite or bradyzoite stage, and previous mRNA sequencing data show that this GRA12-related gene is highly expressed in the merozoite/oocyst stage (39). Our results show that GRA12-related genes TgME49_220890, TgME49_275860, and TgME49_308970 are expressed as IVN membrane-associated GRA proteins that localize to the cyst wall and cyst matrix and influence the development and persistence of chronic stage cyst burdens. In addition, we report a novel quantitative *in vitro* cyst reactivation assay that revealed significant defects in the reactivation of cysts that lacked expression of GRA12-related genes.

## RESULTS

### The GRA12 gene family.

Dense granule organelles secrete GRA proteins that rarely exhibit recognizable domains or homology to other proteins (11, 12). Thus far, at least 57 unique GRAs (GRA1 to GRA25 and GRA28 to GRA59) have been identified, and only four of these GRAs (GRA11, GRA12, GRA20, and GRA21) reside in multigene families that share sequence similarity (26, 40–42). The *Toxoplasma* database (www.toxoDB.org), revealed four genes that share significant homology to GRA12 (TgME49_288650): GRA12A:TgME49_220890, GRA12B:TgME49_275860, GRA12C:TgME49_275850, and GRA12D:TgME49_308970 (see Fig. S1A in the supplemental material). Each of these GRA12-related genes possess a signal peptide, though only one, GRA12A, possessed a distinct predicted transmembrane domain (Fig. S1A). Alignment of the five GRA12-related proteins of the GRA12 family revealed 28 universally conserved amino acids, including four cysteines, indicating a potential shared folding scaffold (Fig. S1B). GRA12-related proteins GRA12A to -D displayed 12% to 28% amino acid identity with GRA12. GRA12A, GRA12B, and GRA12D exhibited peak mRNA expression in the tachyzoite and bradyzoite stages, and in contrast, GRA12C peak mRNA expression was in the merozoite/oocyst stage (39). GRA12C expression in tachyzoite or bradyzoite stages is extremely low, or absent (Fig. S2A and B) (39). Thus, the GRA12-related genes share significant amino acid homology and putative structural similarity (conserved cysteines) but do not share identical patterns of stage-specific expression. These observations support the previous proposal (28) that the GRA12-related genes are probable GRA12 gene paralogs.

10.1128/mSphere.00182-21.1 FIG S1 The GRA12-related gene family. (A) Characteristics of GRA12-related genes determined from ToxoDB data. The percent identity was determined by comparison of each GRA12-related protein with GRA12. (B) Amino acid alignment of GRA12-related proteins. Download FIG S1, TIF file, 1.0 MB. Copyright © 2021 Guevara et al. 2021 Guevara et al. https://creativecommons.org/licenses/by/4.0/ This content is distributed under the terms of the Creative Commons Attribution 4.0 International license.

10.1128/mSphere.00182-21.2 FIG S2 GRA12-related genes are differentially expressed between life stages. (A) Characteristics of GRA12-related genes determined from ToxoDB data. (B) mRNAseq coverage of GRA12-related genes in different *Toxoplasma* stages (from ToxoDB data). Download FIG S2, TIF file, 1.8 MB. Copyright © 2021 Guevara et al. 2021 Guevara et al. https://creativecommons.org/licenses/by/4.0/ This content is distributed under the terms of the Creative Commons Attribution 4.0 International license.

### GRA12-related genes localize to the dense granules and associate with the intravacuolar network membranes in the PV space.

**FIG 1:**
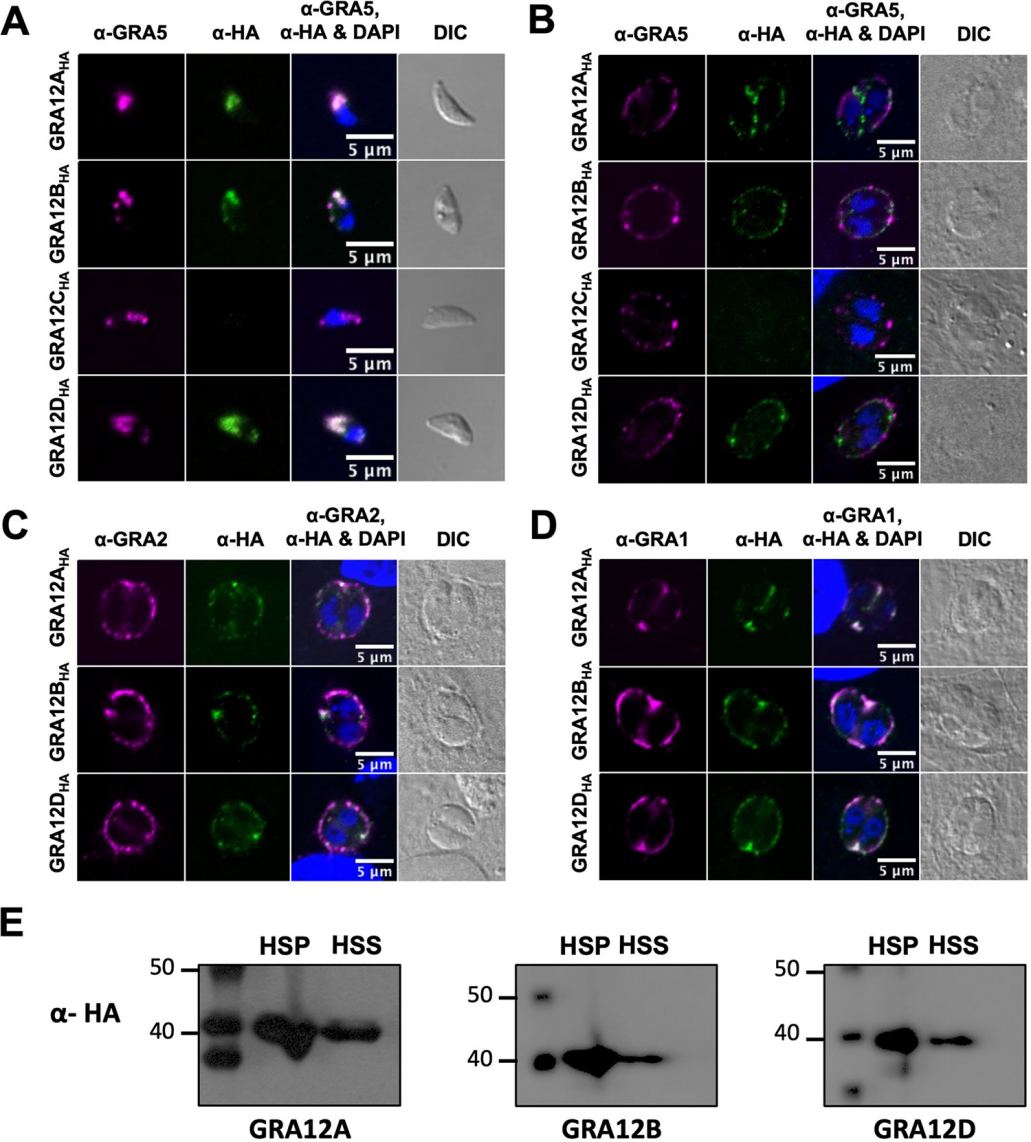
GRA12-related proteins localize to tachyzoite dense granules, are secreted into the PV, and behave as a soluble and transmembrane PV proteins. (A) Extracellular tachyzoites were stained with anti-HA (α-HA) and anti-GRA5 (α-GRA5) antibody. Tachyzoite nuclei were stained with 4′,6-diamidino-2-phenylindole (DAPI). Tachyzoites were located using differential interference contrast (DIC) microscopy and imaged by confocal microscopy. Number (*n*) of tachyzoites analyzed (*n *= 46 for GRA12A, *n *= 39 for GRA12B, *n *= 9 for GRA12C, *n *= 19 for GRA12D). Bars = 5 μm. (B to D) Human foreskin fibroblast (HFF) cells on coverslips were infected with 0.5 MOI tachyzoites of each strain. Vacuoles were located using DIC microscopy, and parasite and host nuclei were stained with DAPI and imaged by confocal microscopy. Vacuoles were stained with anti-HA and anti-GRA5 (B), anti-GRA2 (C), or anti-GRA1 (D). Number (*n*) of vacuoles analyzed, 6 to 42. Bars = 5 μm. (E) PV proteins were fractionated by ultracentrifugation into high-speed pellet (HSP) and high-speed supernatant (HSS) fractions. Proteins were resolved by electrophoresis, transferred to membranes, and probed with anti-HA rabbit antibody specific for HA-tagged GRA12A, GRA12B, or GRA12D, labeled with goat anti-rabbit IgG coupled to peroxidase, and visualized by chemiluminescence.

GRA12 associates with the IVN membranes as a transmembrane protein (26, 28). Given the essential role of GRA12 for resistance to host IFN-γ and successful acute infection, we sought to further characterize GRA12A to -D. The GRA12A to -D genes were deleted (Fig. S3A), and a hemagglutinin (HA) epitope was placed in frame at the C terminus of each GRA12-related gene and then complemented into the endogenous locus in the respective Δ*gra* strain (Fig. S3B). Deletion and complemented strains of GRA12A to -D were confirmed by PCR (Fig. S4A to F), and complemented GRA12-related gene protein expression was confirmed by immunofluorescence (Fig. S4G to I). To confirm that GRA12-related proteins also localized to the dense granule organelles, we stained free tachyzoites with anti-GRA5, a known dense granule marker, and anti-HA (Fig. 1A). GRA12A, GRA12B, and GRA12D colocalized with anti-GRA5, confirming their dense granule localization and GRA designation (Fig. 1A). As expected from mRNA expression data (Fig. S2A), expression of the GRA12C protein was not detected in tachyzoites (Fig. 1A) or in the PV (Fig. 1B), thus further experiments on GRA12C were not pursued. To examine PV localization, we colocalized GRA12A, GRA12B, and GRA12D with anti-GRA5 (PV membrane), anti-GRA2 (IVN membranes), and anti-GRA1 (PV space) (43). GRA12A, GRA12B, and GRA12D did not colocalize with GRA5 at the PV membrane and were not translocated into the host cell (Fig. 1B). Instead, GRA12-related proteins colocalized with GRA2 at the PV periphery with the IVN membranes (Fig. 1C) and with GRA1 in the PV space (Fig. 1D). Since IVN-associated GRAs can associate with and disassociate from the IVN membranes (12), these results suggested that similar to GRA12, GRA12A, GRA12B, and GRA12D preferentially associated with the IVN. To confirm this localization pattern, the PV was fractionated into a high-speed pellet (HSP), which includes PVM and IVN membrane proteins, or a high-speed supernatant (HSS), which includes PV soluble proteins. As expected, GRA12A, GRA12B, and GRA12D were observed in both the HSP and HSS fractions (Fig. 1E), confirming their localization as IVN-associated GRA proteins.

10.1128/mSphere.00182-21.3 FIG S3 GRA12-related gene knockout and complementation strategy. (A) Knockout strategy to insert *HXGPRT* at deleted GRA gene loci by selection with mycophenolic acid (MPA) and xanthine (X). Design of genotype validation PCR is shown. (B) Complementation strategy at the original locus through selection with 6TX (6-thioxanthine). Design of genotype validation PCR is shown. Download FIG S3, TIF file, 1.6 MB. Copyright © 2021 Guevara et al. 2021 Guevara et al. https://creativecommons.org/licenses/by/4.0/ This content is distributed under the terms of the Creative Commons Attribution 4.0 International license.

10.1128/mSphere.00182-21.4 FIG S4 Validation of GRA12-related gene knockouts by PCR and validation of complementation by immunofluorescence or PCR. (A to D) Validation of GRA12-related gene deletion in Δ*gra* parasite mutant strains by amplifying a sequence within the gene (PCR1) and a sequence in the 3’UTR (PCR2) using polymerase chain reaction (PCR). The positive control is the parental strain PruΔ*ku80* (+), the negative control is PruΔ*ku80* without addition of primers (−), and the marker (M) is shown. (A) PCR1 is 379 bp, and PCR2 is 573 bp. (B) PCR1 is 399 bp, and PCR2 is 1,011 bp. (C) PCR1 is 456 bp, and PCR2 is 391 bp. (D) PCR1 is 257 bp, and PCR2 is 717 bp. (E and F) Validation of complemented GRA12C using PCR. The positive control is the parental strain PruΔ*ku80* (+), the negative control is PruΔ*ku80* without addition of primers (−), an additional control is the Δ*gra* strain (Δ), and the marker (M) is shown. (E) PCR1 is 456 bp. (F) PCR5 is 1.8 kb, and PCR6 is 664 bp. (G to I) Validation of complemented HA-tagged GRA12 genes in the endogenous loci. HFFs were infected with Δ*gra* and Δ*gra*::GRA tachyzoites for 24 h, permeabilized with 0.01% saponin, and stained with anti-GRA1 and anti-HA antibody. Samples were imaged by confocal microscopy, and vacuoles were located using differential interference contrast (DIC) microscopy. 4′,6-diamidino-2-phenylindole (DAPI) stains the host and parasite nuclei. Representative images are shown for each strain. Bars, 5 μm. Download FIG S4, TIF file, 1.7 MB. Copyright © 2021 Guevara et al. 2021 Guevara et al. https://creativecommons.org/licenses/by/4.0/ This content is distributed under the terms of the Creative Commons Attribution 4.0 International license.

### GRA12-related gene knockouts exhibit moderate defects in acute virulence.

The deletion of other GRAs that associate with the IVN membranes such as GRA2, GRA4, GRA6, GRA9, and GRA12 does not affect the *in vitro* replication rate (26, 38). While replication of Δ*gra12A* and Δ*gra12D* tachyzoites was not affected in a 45-h replication assay, Δ*gra12B* tachyzoites exhibited a 36% increase in the rate of replication compared to the parental PruΔ*ku80* strain (Fig. S5A). Analysis of individual vacuoles revealed an increase in the percentage of Δ*gra12B* PVs with more than 36 tachyzoites per vacuole (Fig. S5B). This increased vacuolar replication phenotype was associated specifically with GRA12B because the complemented GRA12B strain exhibited the parental strain replication phenotype and individual vacuoles contained a similar number of parasites (Fig. S5A and B).

10.1128/mSphere.00182-21.5 FIG S5 The Δ*gra12B* mutant exhibits increased replication rate, and GRA12-related gene knockout mutants exhibit moderate defects in acute virulence *in vivo*. (A and B) Replication rates of Δ*gra12A*, Δ*gra12B*, Δ*gra12D*, and complemented GRA12B parasites were measured in infected HFF cells in a 45-h assay in comparison to PruΔ*ku80.* (A) Tachyzoites per vacuole were measured in three independent experiments (*n *= 50 vacuoles). Data are shown as means ± SEM. A one-way ANOVA test revealed significance in reactivation [F(4,10) = 3.859, *P *= 0.03], and Student’s *t* test was used to calculate *P* values in comparison to parental control PruΔ*ku80* as follows: *, *P* < 0.05. (B) The percentage of vacuoles (*n *= 150 vacuoles) with 4, 6, 8, 10, 12, 14, 16, 18, or ≥36 parasites per vacuole is shown. (C and D) Δ*gra12A*, Δ*gra12B*, Δ*gra12D* parasite strains were evaluated for virulence lethality in mice compared to PruΔ*ku80*. C57BL/6 female mice were infected with 2 × 10^6^ PruΔ*ku80* (two experiments; *n *= 8), Δ*gra12A* (one experiment; *n *= 4), Δ*gra12B* (two experiments; *n *= 12), or Δ*gra12D* (one experiment; *n *= 4) tachyzoites (C) or with 2 × 10^5^ PruΔ*ku80* (two experiments; *n *= 8), Δ*gra12A* (two experiments; *n *= 8), Δ*gra12B* (two experiments; *n *= 8), or Δ*gra12D* (two experiments; *n *= 8) tachyzoites (D) by intraperitoneal injection, and mice were monitored for 30 days. *P* value was calculated by a log rank Mantel-Cox test, and *P* < 0.05 was considered significant. Statistical significance: **, *P* < 0.01; ***, *P* < 0.005; NS, not significant. Download FIG S5, TIF file, 1.1 MB. Copyright © 2021 Guevara et al. 2021 Guevara et al. https://creativecommons.org/licenses/by/4.0/ This content is distributed under the terms of the Creative Commons Attribution 4.0 International license.

Since GRA12 is a major virulence factor required for parasite survival *in vivo*, we evaluated the Δ*gra12A*, Δ*gra12B*, and Δ*gra12D* mutants for virulence lethality in mice. Δ*gra12A*, Δ*gra12B*, and Δ*gra12D* mutants and the parental PruΔ*ku80* strain were virulent, and mice succumbed after intraperitoneal infection with 2 × 10^6^ tachyzoites (Fig. S5C). At a lower infection dose of 2 × 10^5^ tachyzoites, the Δ*gra12B* and Δ*gra12D* mutants exhibited a detectable defect in acute virulence (Fig. S5D). In contrast to Δ*gra12* parasites which are avirulent after infection with 2 × 10^7^ tachyzoites (26), the deletion of GRA12-related genes induced moderate virulence defects rather than a major defect in acute virulence. Therefore, these findings further support the hypothesis that GRA12A, GRA12B, and GRA12D have likely diverged from GRA12 and have developed different functions, a common occurrence among gene paralogs.

### GRA12-related genes are expanded in Coccidians that form cysts in the brain.

BLASTP searching the *Toxoplasma* database (www.toxoDB.org) (41) revealed that GRA12-related genes are also present in other Coccidians (*Hammondia*, *Neospora*, *Cystoiospora*, *Eimeria*, *Cyclospora*, and *Sarcocystis* species) (Fig. S6A). However, while universally conserved amino acids were shared between all of the Coccidian GRA12-related genes (Fig. S6B), the absolute number of distinct GRA12-related genes varied between the different Coccidians (Fig. S6A). In Coccidian species that are not recognized to form brain cysts (*Eimeria*, *Cyclospora*, *Sarcocystis*, and *Cystoisospora*), two to four GRA12-related genes were present, and in *Cystoisospora* with four predicted GRA12-related genes, the GRA12B-related gene exhibited extremely low similarity (only 6%) to GRA12B expressed by brain cyst-forming Coccidians (Fig. S6A). In contrast, in Coccidians that are recognized to form brain cysts (*Neospora*, *Hammondia*, and *Toxoplasma*), four or five GRA12-related genes were present, and each gene exhibited moderate to high gene similarity (45% to 94%) in comparison to the corresponding *Toxoplasma* gene. These observations suggested that GRA12-related genes were expanded in brain cyst-forming Coccidians, indicating their possible function during the chronic stages of infection.

10.1128/mSphere.00182-21.6 FIG S6 GRA12-related genes are expanded in brain cyst-forming Coccidians and are localized within the developing 6-h-old cyst. (A) A summary of the GRA12, GRA12A, GRA12B, GRA12C, and GRA12D genes found in Coccidians (data from ToxoDB). The percentage of identical amino acids of a GRA gene across Coccidians relative to *Toxoplasma* is displayed for each gene. (B) The universally conserved GRA12 amino acids found in Eimeria tenella, *Cyclospora*, *Neospora*, *Hammondia*, and *Toxoplasma*. (C) Infected HFF cells on coverslips were treated under bradyzoite-inducing conditions for 6 h to differentiate *in vitro* cysts. Cysts containing GFP^+^ bradyzoites were located using DIC microscopy and imaged by confocal microscopy. Cysts were stained with DBA and anti-HA antibody. Panels show DAPI and GFP, DBA and GFP, DBA, anti-HA, DBA and anti-HA, and DIC. Number (*n*) of vacuoles analyzed, 11 to 13. The occurrence of the phenotype was 100% observed in each vacuole for GRA12A, GRA12B, and GRA12D. Bars = 5 μm. Download FIG S6, TIF file, 2.0 MB. Copyright © 2021 Guevara et al. 2021 Guevara et al. https://creativecommons.org/licenses/by/4.0/ This content is distributed under the terms of the Creative Commons Attribution 4.0 International license.

### In immature cysts, GRA12-related proteins localize to the developing cyst wall and to the cyst matrix.

**FIG 2:**
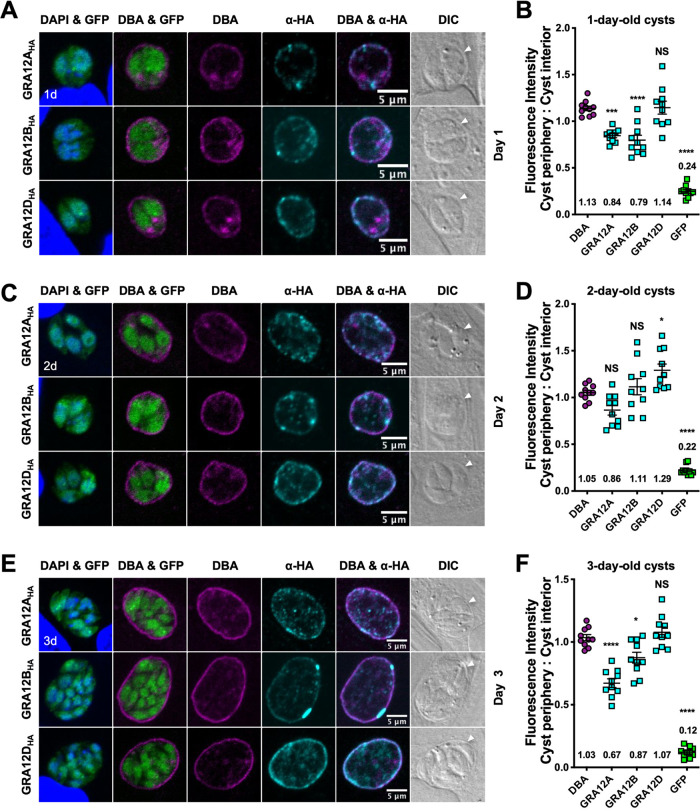
GRA12-related proteins localized to the cyst periphery and cyst matrix in immature cysts. (A, C, and E) HFF cells were infected with complemented GRA12-related HA-tagged protein strains, and *in vitro* cysts were differentiated for 1 day (1d) (A), 2 days (2d) (C), or 3 days (3d) (E). Cysts were located using DIC microscopy and imaged by confocal microscopy. The presence of bradyzoites was verified by locating parasite nuclei using DAPI stain and verifying that each parasite nucleus was surrounded by expression of cytosolic GFP (GFP^+^ bradyzoites). DAPI stains the host and parasite nuclei. Cysts were stained with DBA and anti-HA antibody. Panels show DAPI and GFP, DBA and GFP, DBA, anti-HA, DBA and anti-HA, and DIC (cyst periphery indicated by white arrowhead). Number (*n*) of cysts analyzed, 10. Bars = 5 μm. (B, D, and F) Fluorescence intensities of DBA, GRA12A, GRA12B, GRA12D, and GFP were measured at the cyst periphery and within the cyst (cyst interior) (*n *= 10 cysts) at 1 day (B), 2 days (D), or 3 days (F). Data plotted as the ratio mean of fluorescence intensity at the cyst periphery to the cyst interior ± standard error of the mean (SEM) (error bar). The numerical ratio for the mean fluorescence intensity is labeled. A one-way ANOVA test was used to reveal significance in mean fluorescence intensity [F(4, 36) = 68.30 at 1 day, 55.35 at 2 days, 115.5 at 3 days, *P* < 0.0001], and then a Tukey test was used to calculate *P* values in pairwise comparison to DBA and shown as follows: *, *P* < 0.05; ***, *P* < 0.001; ****, *P* < 0.0001; NS, not significant.

Previously, we tracked the localization of IVN GRA proteins GRA2, GRA4, GRA6, GRA9, and GRA12 during cyst development to different layers in the cyst wall and to distinct patterns in the cyst matrix (30). To track localization of GRA12-related proteins in cysts, we used high pH shift to *in vitro* differentiate tachyzoites inside PVs into encysted bradyzoites (44), DBA to stain the cyst wall (35, 36), and bradyzoite stage-specific cytosolic expression of green fluorescent protein (GFP^+^) to localize bradyzoites (30). By 6 h postdifferentiation, GFP^+^ bradyzoites were visible within the developing cyst (Fig. S6C). DBA staining and GRA12A, GRA12B, and GRA12D proteins were observed within recently differentiated young cysts (Fig. S6C). By 1 day and 2 day postdifferentiation, GRA12A, GRA12B, and GRA12D localized to the vacuole periphery with DBA in immature cysts (Fig. 2A to D), where the cyst wall develops. Differences in localization patterns between the GRA12-related proteins emerged by 3 days after differentiation (Fig. 2E and F): GRA12A was interspersed throughout the developing cyst wall, GRA12B was seen as bright puncta at the cyst wall, and GRA12D intensely stained the cyst wall. Like DBA stain, GRA12D was preferentially observed at the developing cyst periphery (Fig. 2A to F), suggesting its possible involvement in cyst wall formation. The localization of GRA12A, GRA12B, and GRA12D with the developing cyst wall in immature cysts suggests their potential role in shaping the PV into a cyst.

### In mature cysts, GRA12-related proteins localize in different patterns in the cyst matrix and to different layers in the cyst wall.

**FIG 3:**
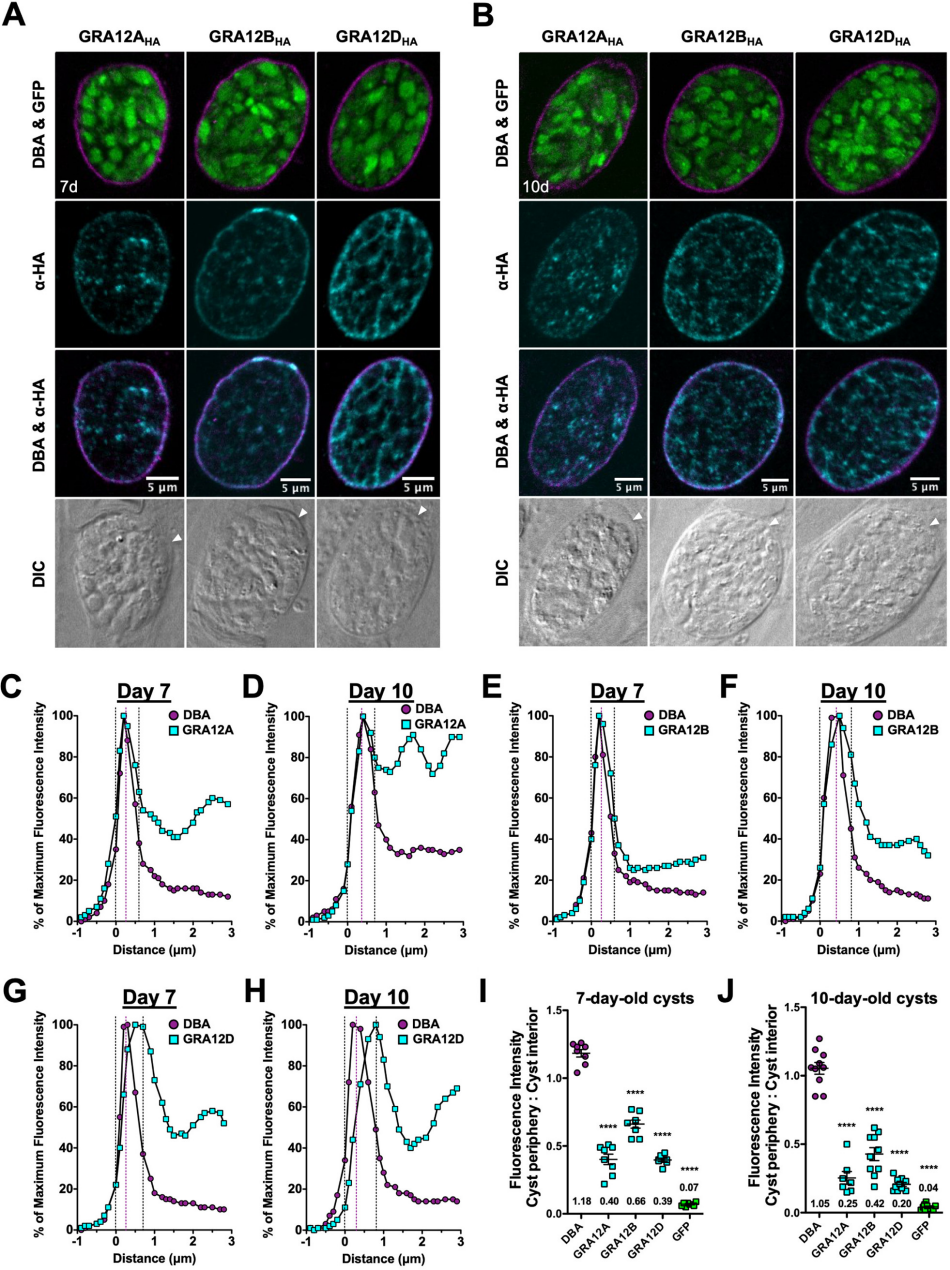
GRA12-related proteins localize to the cyst wall and cyst matrix in mature cysts. (A and B) HFF cells were infected with complemented GRA12-related HA-tagged protein strains, and *in vitro* cysts were differentiated for 7 days (A) or 10 days (B). Cysts containing GFP^+^ bradyzoites were located using DIC microscopy and imaged by confocal microscopy. Cysts were stained with DBA and anti-HA antibody (α-HA). Panels show DBA and GFP, anti-HA, DBA and anti-HA, and DIC (cyst periphery indicated by white arrowhead). Number (*n*) of cysts analyzed, 8 to 12. Bars = 5 μm. (C to H) Fluorescence intensity profiles of representative 7- and 10-day-old cysts, shown in panels A and B, were generated to quantify the location of DBA relative to GRA12A (C and D), GRA12B (E and F), or GRA12D (G and H) at the cyst wall. Dotted black lines define the cyst wall region. The dotted purple line indicates the middle of the cyst wall, which corresponds to the peak DBA fluorescence intensity. (I and J) Fluorescence intensities of DBA, GRA12A, GRA12B, GRA12D, and GFP were measured at the cyst periphery and within the cyst (cyst interior) at 7 days (*n *= 8 cysts) (I) or 10 days (*n *= 7 to 10 cysts) (J). Data plotted as the ratio mean of fluorescence intensity at the cyst periphery to the cyst interior ± SEM. The numerical ratio for the mean fluorescence intensity is labeled. A one-way ANOVA test was used to reveal significance in mean fluorescence intensity [F(4, 28) = 223.4 at 7 days, F(4,42) = 134.4 at 10 days, *P* < 0.0001], and then a Tukey test was used to calculate *P* values in pairwise comparison to DBA as follows: ****, *P* < 0.0001.

Next, we examined the location of GRA12-related proteins in mature cysts after differentiation for 7 or 10 days. GRA12B and GRA12D occupied the cyst matrix spaces between the bradyzoites, while puncta of GRA12A were visible in the cyst matrix (Fig. 3A and B). GRA12A, GRA12B, and GRA12D were also localized with the cyst wall (Fig. 3A and B). To quantitatively assess the location(s) of GRA12-related proteins in the cyst wall, we measured the cyst fluorescence intensity profiles for GRA12-related proteins and DBA as previously described (30). The DBA-stained cyst wall of a mature cyst is composed of two layers, an outer layer that is more compact and a loose inner layer that faces the cyst matrix (34). The cyst wall region in 7- and 10-day-old cysts was measured as previously described (30). The cyst wall region is highlighted by dotted vertical black lines, and the peak of DBA fluorescence, a cyst wall marker, is indicated by a dotted magenta line (Fig. 3C to J). The fluorescence intensity peaks of GRA12A, GRA12B, and GRA12D overlapped with the DBA fluorescence intensity peak. The intensity peaks of GRA12A (Fig. 3C and D) and GRA12B (Fig. 3E and F) were similar to the peak of DBA, suggesting the presence of GRA12A and GRA12B throughout the cyst wall. In contrast, the intensity peak of GRA12D was shifted to the right (toward the cyst interior) compared to the DBA peak (Fig. 3G and H), indicative of GRA12D being present in the less dense inner layer of the cyst wall. In comparison to DBA, GRA12A, GRA12B, and GRA12D exhibited significant decreases in fluorescent intensity at the cyst periphery relative to the cyst interior in 7-day (Fig. 3I) and 10-day-old cysts (Fig. 3J), and this measurement revealed that these GRA12-related proteins were also prominent in the cyst interior.

### GRA12-related proteins colocalize with GRA2 in the cyst matrix and at the cyst wall.

**FIG 4:**
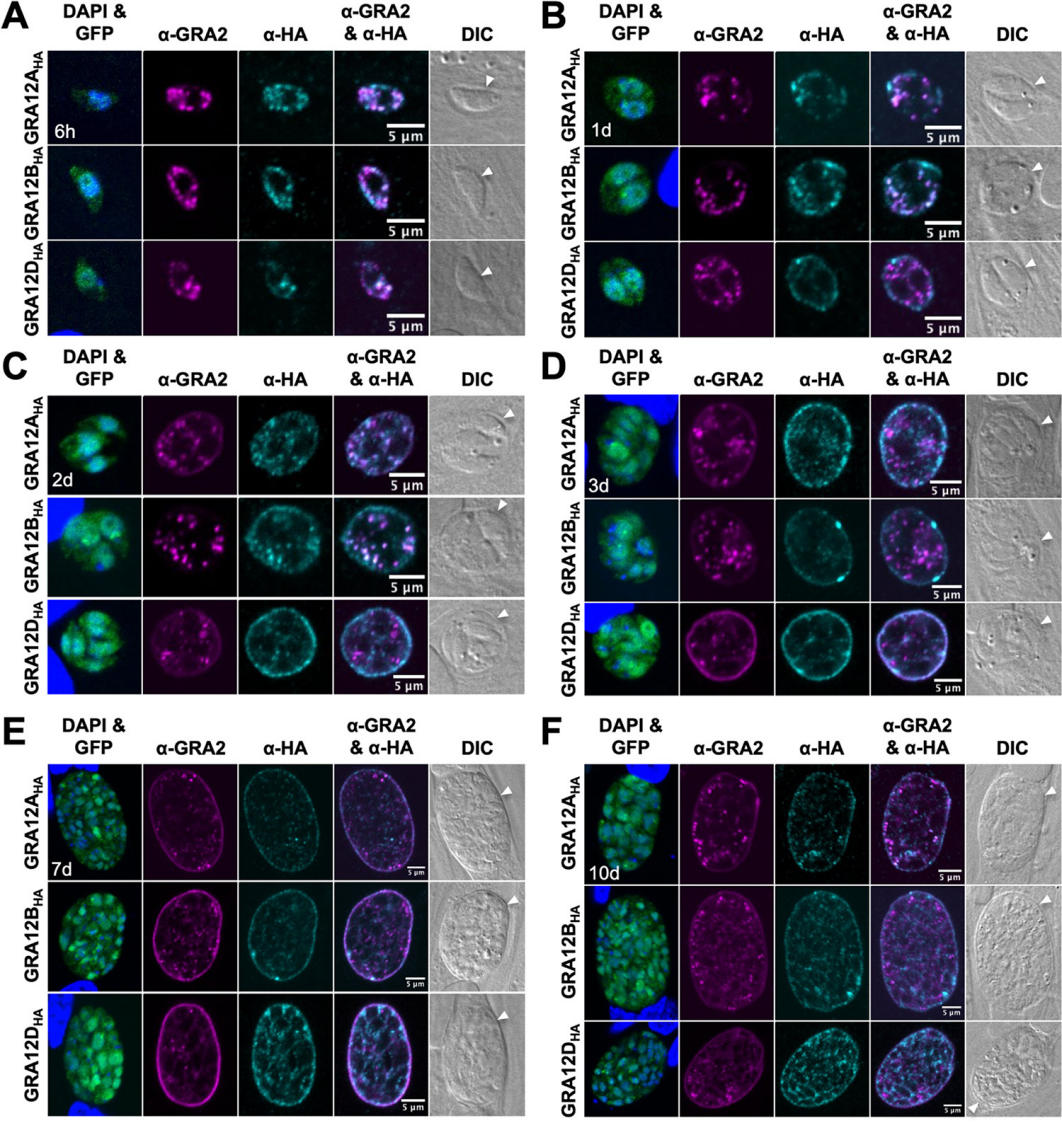
GRA12-related proteins colocalize with GRA2 in the cyst matrix and at the cyst wall. HFF cells were infected with complemented GRA12-related HA-tagged protein strains, and *in vitro* cysts were differentiated for 6 h (A), 1 day (B), 2 days (C), 3 days (D), 7 days (E), or 10 days (F). Cysts containing GFP^+^ bradyzoites were located using DIC microscopy and imaged by confocal microscopy. DAPI stained host and parasite nuclei. Cysts were stained with anti-GRA2 and anti-HA antibody. Panels show DAPI and GFP, anti-GRA2, anti-HA, anti-GRA2 and anti-HA, and DIC (cyst periphery/wall indicated by white arrowhead). GRA2 colocalized with GRA12-related proteins in 100% of cysts evaluated at 6 h (*n *= 11 to 13), with GRA12A and GRA12B in 100% of cysts evaluated at 1 day (*n *= 13 to 16) and 2 days (*n *= 6 to 12), and with GRA12D in 100% of cysts evaluated at 3 days (*n *= 17). Occurrence of GRA2 colocalization phenotype was 100% in all cysts analyzed, 1 day (*n *= 9 to 16), 2 days (*n *= 7 to 12), 3 days (*n *= 17 to 22), 7 days (*n *= 10 to 24), and 10 days (*n *= 12 to 15). Bars = 5 μm.

GRA2 is essential for the formation of the tubular membranes of the IVN within the PV during an acute infection and for the organization of the cyst matrix during a chronic infection (14, 30). We previously colocalized GRA2 with the GRA12 protein throughout cyst development (30). To determine whether GRA12-related proteins also colocalize with GRA2, we tracked the colocalization of GRA2 with GRA12A, GRA12B, or GRA12D in *in vitro* cysts differentiated for 6 h, 1 day, 2 days, 3 days, 7 days, or 10 days. At 6 h, the GRA12-related proteins colocalized with GRA2 as puncta within the differentiating vacuole, which indicates an association with GRA2 (Fig. 4A). In 1- and 2-day-old cysts, GRA12A and GRA12B were localized inside the cyst PV and at the periphery where the cyst wall is developing, and GRA12A and GRA12B colocalized with GRA2 inside the cyst PV (Fig. 4B and C). Interestingly, GRA12D was seen predominantly at the periphery and was not colocalized with GRA2 (Fig. 4B and C). These results suggested that early in differentiation (at 6 h), GRA12A, GRA12B, and GRA12D were associated with GRA2, then like other IVN GRAs that traffic with GRA2, disassociated from GRA2 at the time they become membrane bound (28, 45). In 3-day-old cysts, GRA2 transitions from inside the cyst to the cyst wall, and this transition correlates with initiation of the organization of the cyst matrix (31). At 3 days after differentiation, GRA12D was colocalized with GRA2 at the cyst wall and in the cyst matrix (Fig. 4D), and GRA12A and GRA12B were seen at the cyst wall as bright puncta, which were particularly large for GRA12B (Fig. 4D). These results demonstrated that significant interactions occur between GRA12-related proteins and GRA2 as the cyst develops. Whether these interactions are specific to the IVN or to GRA2 is not yet known.

In mature cysts, GRA2 has been observed at the cyst wall (30, 43). Consistent with this localization, we observed that GRA12-related proteins colocalized at the cyst wall with GRA2 in 7- and 10-day-old cysts (Fig. 4E and F). The fluorescence intensity peak of GRA12A, GRA12B, and GRA12D overlapped with GRA2 (Fig. S7A to F). In addition, the fluorescence intensity peak of GRA12D was shifted slightly to the left of the GRA2 fluorescence intensity peak (Fig. S7F), further supporting the presence of GRA12D in the inner layer of the cyst wall in mature cysts.

10.1128/mSphere.00182-21.7 FIG S7 GRA12-related proteins colocalize with GRA2 in the cyst matrix and with GRA2 at the cyst wall. (A to F) Fluorescence intensity profiles of representative cysts shown in Fig. 4E and F were generated to quantify the location of GRA12A, GRA12B, or GRA12D relative to GRA2 at the cyst wall at day 7 (A to C) and day 10 (D to F). The dotted black vertical lines define the cyst wall region. The outer boundary is the first point less than 50% GRA2, and the inner boundary is the first point less than 50% GFP. (G) Quantification of cooccurrence and correlation coefficients between GRA2 and GRA12A or GRA12B or GRA12D were measured at 6 h, 1 day, 2 days, 3 days, 7 days, and 10 days to evaluate colocalization. Pearson’s correlation coefficient (PCC) measures whether a linear correlation of fluorescent intensities exists between the magenta signal and the cyan signal at every pixel (PCC of 1 indicates a perfect correlation). Manders’ coefficients for each channel (M1 and M2) incorporate the relationship between intensity and concentration. Spearman’s rank correlation coefficients (SRCC) measure correlations that may not be linear by using pixel intensity rank instead of value which is measured by PCC. Data shown are means ± standard deviations. Number of cysts analyzed for each strain and time point (*n *= 7). Download FIG S7, TIF file, 1.8 MB. Copyright © 2021 Guevara et al. 2021 Guevara et al. https://creativecommons.org/licenses/by/4.0/ This content is distributed under the terms of the Creative Commons Attribution 4.0 International license.

To quantitatively assess the colocalization between GRA2 and the GRA12-related proteins, measurements of cooccurrence and correlation coefficients were measured in 6-h, 1-day, 2-day, 3-day, 7-d, and 10-day-old cysts. Pearson’s correlation coefficient (PCC) measures whether a linear correlation of fluorescent intensities exists between the two signals at every pixel. Spearman’s rank correlation coefficient (SRCC) measures correlations that may not be linear by using pixel intensity rank instead of value, which is measured by PCC. A strong correlation, as indicated by a coefficient greater than 0.60, was seen at 6 h, 1 day, and 2 days for GRA12A and GRA12B, and at 3 days for GRA12D (Fig. S7G). A moderate correlation, as indicated by a coefficient between 0.60 and 0.50, was observed at 7 days for GRA12A and GRA12D and at 10 days for GRA12A (Fig. S7G). Manders’ coefficient evaluates the relationship between intensity and concentration. The individual Manders’ coefficients (M1 and M2) revealed that the majority of GRA2 signal (M1) overlapped with the GRA12-related proteins (greater than 0.92) and that not all of the GRA12-related protein signal (M2) overlapped with GRA2 (Fig. S7G), which supports the fluorescent patterns seen in the captured images. For a control, Costes’ randomization was performed on every image, and the Mander’s coefficient exceeded 95% of the scrambled values, which supports the conclusion that the cooccurrence observed between GRA2 and GRA12-related proteins was not due to random chance.

### GRA12-related gene mutants exhibit defects in chronic infection.

**FIG 5:**
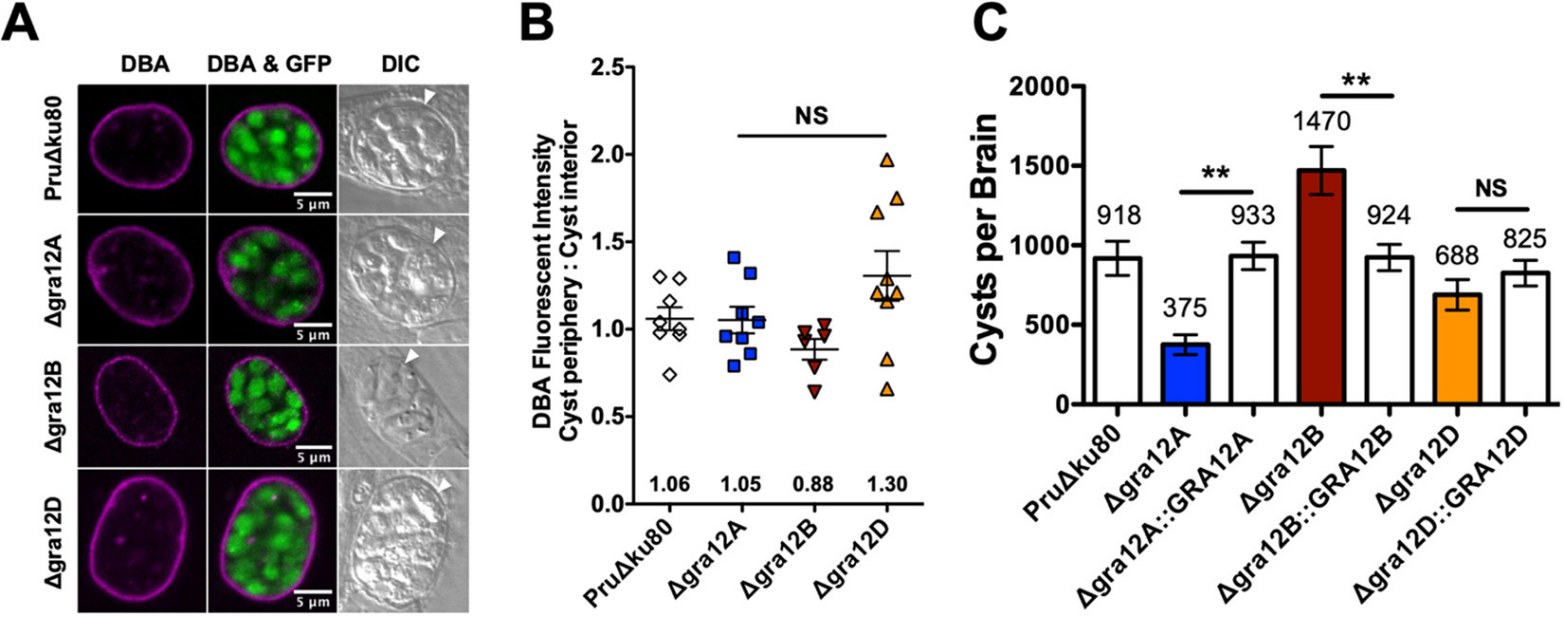
GRA12-related gene knockout mutants differentiate normally *in vitro* and exhibit defects in cyst burdens *in vivo*. (A) HFFs were infected with the indicated strains, and cysts differentiated *in vitro* cysts for 7 days. Cysts containing GFP^+^ bradyzoites were located using DIC microscopy and imaged by confocal microscopy. Cysts were stained with DBA. Number (*n*) of cysts analyzed, 6 to 8. Bars = 5 μm. (B) Cysts from each strain were analyzed to determine the ratio of DBA staining intensity at the cyst periphery relative to the cyst interior. Data were plotted as the average ratio mean of fluorescence intensity at the cyst periphery to the cyst interior ± SEM for each strain (*n *= 6 to 8 cysts). A one-way ANOVA analysis showed no significance in the mean fluorescence intensity ratio [F(3, 27) = 2.91, *P* = 0.0527]. (C) C57BL/6 mice were infected with 2 × 10^2^ tachyzoites by intraperitoneal injection, and brain cysts were measured 3 weeks after infection. Data shown are cumulative results from two to six independent experiments for each strain tested and are shown as means ± SEM. PruΔ*ku80* (three experiments; *n *= 12 mice), Δ*gra12A* (two experiments; *n *= 8 mice), Δ*gra12A*::GRA12A (two experiments; *n *= 6 mice), Δ*gra12B* (two experiments; *n *= 8 mice), Δ*gra12B*::GRA12B (four experiments; *n *= 13 mice), Δ*gra12D* (six experiments; *n *= 21 mice), Δ*gra12D*::GRA12D (three experiments; *n *= 10 mice). At least 10% of each brain was scanned for GFP^+^ cysts. A one-way ANOVA test revealed significance in the number of cysts per brain [F(6, 71) = 7.5, *P* < 0.0001], and a Tukey test was used to calculate *P* values in comparison to parental control PruΔ*ku80* and the respective complemented strain as follows: **, *P* < 0.01; NS, not significant.

The Δ*gra12A*, Δ*gra12B*, and Δ*gra12D* mutants stage differentiated *in vitro* into bradyzoites expressing green fluorescent protein (GFP^+^) under the control of the bradyzoite-specific lactate dehydrogenase gene 2 (LDH2) promoter (46, 47), and 7-day-old cysts possessed an intact cyst wall structure that was visible with DBA stain, similar to parental PruΔ*ku80* (Fig. 5A). To measure any defects in the development of the cyst wall, we measured the DBA fluorescence intensity ratio (cyst periphery/cyst interior). The DBA stain was measured at the cyst periphery, which reflects CST1 molecules at the cyst wall compared to the cyst interior, which reflects CST1 molecules not yet at the cyst wall. In contrast with deletion of GRA12 (26), no defects were observed in the development of the *in vitro* cyst wall in cysts that lacked expression of a GRA12-related gene (Fig. 5B).

We evaluated Δ*gra12A*, Δ*gra12B*, and Δ*gra12D* mutants for their ability to establish chronic infection in mice. Mice were infected intraperitoneally with 200 tachyzoites, and brain cysts were measured 3 weeks after infection. Chronic stage cyst burdens were normal in mice infected with the Δ*gra12D* mutant (Fig. 5C). In contrast, a significant decrease in cyst burden (cyst reduction of 59%) was observed after infection with the Δ*gra12A* mutant. Surprisingly, a significant increase in cyst burden (cyst increase of 60%) was observed after infection with the Δ*gra12B* mutant (Fig. 5C).

### GRA12-related gene mutants exhibit defects in cyst maintenance and cyst reactivation.

**FIG 6:**
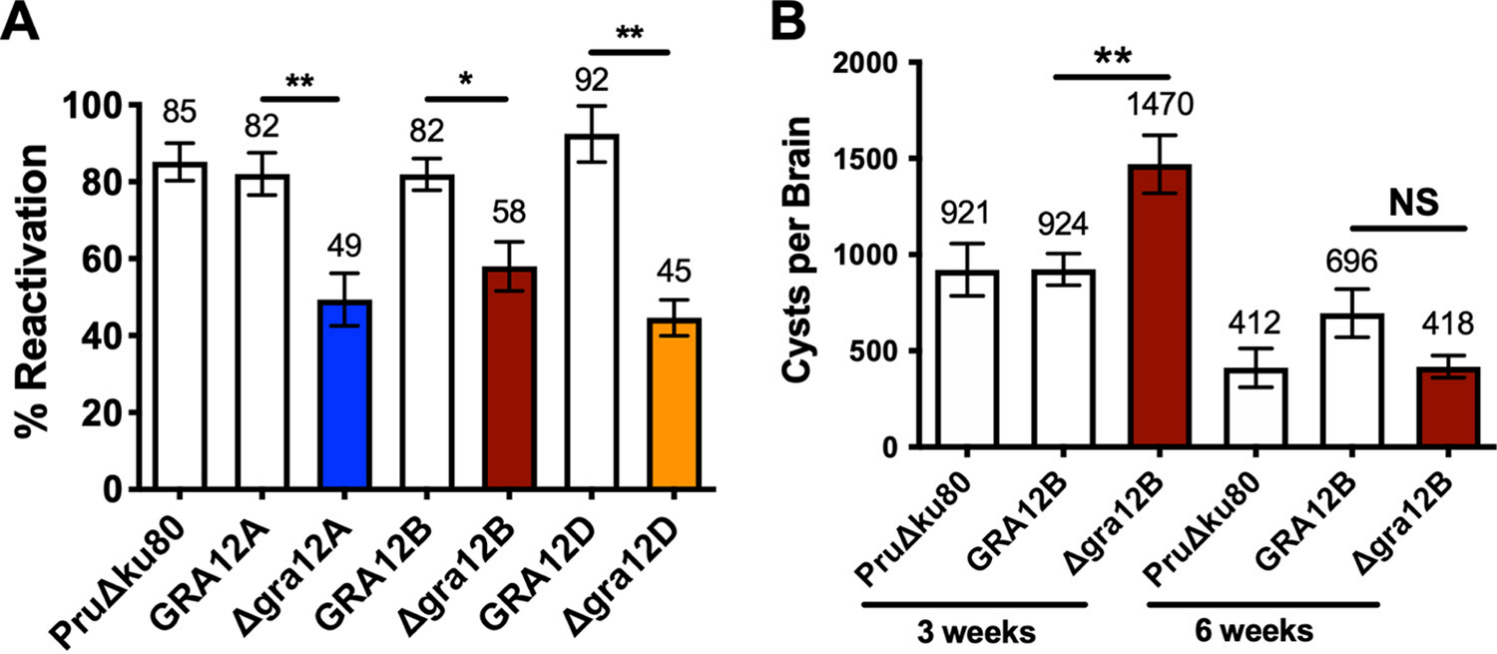
GRA12-related gene knockout mutants exhibit reduced cyst maintenance and cyst reactivation. (A) Reactivation assays were performed using *in vitro* cysts differentiated for 3 days after HFF cell cultures were infected with 2 × 10^2^ tachyzoites of parental PruΔ*ku80*, Δ*gra12A*::GRA12A or Δ*gra12A*, Δ*gra12B*::GRA12B or Δ*gra12B*, and Δ*gra12D*::GRA12D or Δ*gra12D*. Percent reactivation (mean ± SEM) from three independent experiments is shown. A one-way ANOVA test revealed significance in reactivation [F(6, 20) = 8.642, *P* = 0.0001], and a Tukey test was used to calculate *P* values in comparison to parental control PruΔ*ku80* and the respective complemented strain as follows: *, *P* < 0.05; **, *P* < 0.01. (B) C57BL/6 female mice (*n *= 6 to 13) were infected i.p. with 2 × 10^2^ tachyzoites of PruΔ*ku80*, Δ*gra12B*, or Δ*gra12B*::GRA12B strain, and brain cyst burdens were measured 3 and 6 weeks postinfection (mean ± SEM). Data shown are cumulative results from two to four independent experiments for each strain. At 3 weeks, PruΔ*ku80* (two experiments, *n *= 8), Δ*gra12B* (two experiments, *n *= 8), and GRA12B (four experiments, *n *= 13) strains are shown. At 6 weeks, PruΔ*ku80* (two experiments, *n *= 6), Δ*gra12B* (two experiments, *n *= 6), and GRA12B (three experiments, *n *= 10) strains are shown. A one-way ANOVA test revealed significance in reactivation [F(5, 45) = 9.909, *P* < 0.0001], and a Tukey test was used to calculate *P* values in comparison to parental control PruΔ*ku80* and the respective complemented strain as follows: **, *P* < 0.01; NS, not significant.

Cyst reactivation is the process whereby bradyzoites encased within cysts dedifferentiate into tachyzoites, and parasites escape from the cyst to invade and replicate in new host cells, causing a reactivation of acute infection (48). To investigate whether GRA12-related gene mutants exhibit defects in cyst reactivation *in vitro*, we developed a quantitative assay to measure individual cyst reactivation events as PFU. Multiple human foreskin fibroblast (HFF) cell cultures were infected with 200 tachyzoites of Δ*gra12A*, Δ*gra12B*, *or* Δ*gra12D* mutants or their corresponding complemented strain and the parental PruΔ*ku80* strain for 3 h. After infection, one half of the cultures were differentiated to induce bradyzoites and *in vitro* cysts, while the other half of the cultures were entered directly into an 11-day PFU assay. After 3 days of differentiation, the *in vitro* cyst cultures were removed from differentiation conditions and rinsed to remove differentiation medium, and the cultures were entered into an 11-day PFU assay. We measured the percentage of cysts that reactivated using the formula (PFU from reactivated cysts/total initial PFU × 100). Δ*gra12A*, Δ*gra12B*, and Δ*gra12D* cysts exhibited a significant decrease in the percentage of PFU that reactivated in comparison with their respective complemented strain and the parental PruΔ*ku80* strain (Fig. 6A).

In addition to reduced reactivation efficiency, the Δ*gra12A* mutant displayed smaller plaques after reactivation (Fig. S8A and B). To determine whether we captured all of the Δ*gra12A* cyst reactivation events as PFU after 3 days of cyst differentiation, we increased the length of PFU development from 11 days to 13, 15, and 17 days (Fig. S8C to E). There was a slight increase in the number of PFU detected with increased time to detect PFU (Fig. S8C). Furthermore, after Δ*gra12A* cyst reactivation occurred, the plaque size steadily increased with time (Fig. S8D and E). All together, these results suggest that Δ*gra12A* cysts exhibit two distinct reactivation defects, a reduced reactivation frequency and a delay in reactivation. Even though the Δ*gra12B* and Δ*gra12D* mutants showed a decrease in percentage reactivation (Fig. 6A), we did not detect reduced plaque size after reactivation of 3-day-old *in vitro* Δ*gra12B* and Δ*gra12D* cysts (Fig. S8A and B). These results suggest that Δ*gra12B* and Δ*gra12D* cysts exhibit a reduced reactivation frequency that does not delay reactivation.

10.1128/mSphere.00182-21.8 FIG S8 Reactivated plaques of GRA12-related gene knockout mutants and a time course of Δ*gra12A in vitro* cyst reactivation. (A and B) Reactivation assays were performed using *in vitro* cysts differentiated for 3 days after HFF cell cultures were infected with 2 × 10^2^ tachyzoites of parental PruΔ*ku80*, Δ*gra12A*::GRA12A, Δ*gra12A*, Δ*gra12B*::GRA12B, Δ*gra12B*, Δ*gra12D*::GRA12D, or Δ*gra12D*. (A) The reactivated plaques (*n *= 50) were measured for PFU size (mean ± SEM) in four independent experiments. A one-way ANOVA test revealed significance in PFU size [F(6, 84) = 6.825, *P* < 0.0001], and a Tukey test was used to calculate *P* values in comparison to parental control PruΔ*ku80* and the respective complemented strain as follows: *, *P* < 0.05; ****, *P *< 0.0001; NS, not significant. (B) Representative PFU are shown for each strain: parental PruΔ*ku80*, Δ*gra12A*::GRA12A, Δ*gra12B*::GRA12B, Δ*gra12D*::GRA12D, Δ*gra12A*, Δ*gra12B*, and Δ*gra12D* (bars = 100 pixels). (C to E) HFF cells were infected with 2 × 10^2^ tachyzoites of Δ*gra12A* or Δ*gra12A*::GRA12A, and *in vitro* cysts were differentiated for 3 days. Cysts were reactivated, and PFU were allowed to develop for an additional 11, 13, 15, or 17 days. HFF cells were stained with Coomassie blue to reveal plaques. (C) The percent cyst reactivation was measured at 14, 16, 18, or 20 days postinfection (mean ± SEM). A one-way ANOVA test revealed significance in reactivation [F(7,22) =7.636, *P* = 0.0001], and Student’s *t* test was used to calculate *P* values in comparison to the complemented strain as follows: *, *P* < 0.05; **, *P* < 0.01. (D) Reactivated PFU size (*n *= 50 plaques per flask) was measured from three independent experiments (*n *= 2 or 3 flasks per experiment) for the Δ*gra12A* strain (mean ± SEM). A one-way ANOVA test revealed significance in plaque area [F(3,31) =9.693, *P* = 0.0001], and a Tukey test was used to calculate *P* values in comparison to 14 days postinfection as follows: **, *P* < 0.01; ***, *P* < 0.005; NS, not significant. (E) Representative reactivated PFU are shown for Δ*gra12A* at 14, 16, 18, or 20 days postinfection (bars = 100 pixels). Download FIG S8, TIF file, 0.8 MB. Copyright © 2021 Guevara et al. 2021 Guevara et al. https://creativecommons.org/licenses/by/4.0/ This content is distributed under the terms of the Creative Commons Attribution 4.0 International license.

Since we observed an increase in cyst burden in the Δ*gra12B* mutant (Fig. 5C), we evaluated the persistence of Δ*gra12B* cysts over time in comparison to parental PruΔ*ku80* and complemented GRA12B strains. Mice were infected with 200 tachyzoites of the Δ*gra12B* mutant, complemented GRA12B, or PruΔ*ku80* strain for 3 or 6 weeks, and then cyst burdens were evaluated. The increased Δ*gra12B* cyst burdens observed at 3 weeks after infection were not observed at 6 weeks after infection (Fig. 6B). Furthermore, at 6 weeks postinfection, the Δ*gra12B* mutant maintained only 28% of cysts present at 3 weeks, whereas the complemented strain maintained 75% of cysts and the parental PruΔ*ku80* strain maintained 45% of cysts. These results suggest that Δ*gra12B* cysts were not efficiently maintained or that these cysts were more effectively cleared by the host.

## DISCUSSION

While many *Toxoplasma* GRA secreted effectors have been characterized to provide significant virulence and survival functions for tachyzoites and the PV (26, 49–51), the role of GRAs in the cyst stages of chronic infection are less well known. It is remarkable that the majority of proteins that are found associated with the cyst wall are GRA proteins (30–34). Deletion of the cyst wall protein (CST1) changes the landscape of the cyst wall and as a result compromises cyst wall stability, which disrupts cyst maintenance and is evidence that the cyst wall is a prominent cyst structure that maintains parasite viability during chronic infection (36). In view of the fact that parasites released from ruptured cysts are cleared by the immune system (52), it has been hypothesized that the cyst wall provides a barrier to cyst clearance. Here, we investigated the possible functions of a family of GRA12-related genes in acute and chronic infection. Table 1 summarizes the major findings and phenotypes linked to a family of GRA12-related genes. It is noteworthy that while GRA12 is a major virulence factor of acute infection (26, 27), only moderate changes in virulence were associated with certain GRA12-related genes. Our findings suggest that a family of GRA12-related genes provides chronic stage functions that affect cyst burdens and cyst reactivation.

**TABLE 1:** Phenotypes of GRA12-related genes during acute and chronic infection^*a*^

Strain	Acute infection			Chronic infection	
	Replication rate of tachyzoites	% survival (2 × 10^6^)	% survival (2 × 10^5^)	Cyst burden (2 × 10^2^)	Reactivation rate (2 × 10^2^)
PruΔ*ku80*	14 ± 1.5	0.0	12.5	918 ± 107	85 ± 4.8
GRA12A	ND	ND	ND	933 ± 86	82 ± 5.4
Δ*gra12A*	13 ± 0.3	0.0	37.5	375 ± 63 ↓	49 ± 6.8 ↓
GRA12B	13 ± 1.7	ND	ND	924 ± 82	82 ± 4.1
Δ*gra12B*	19 ± 0.5 ↑	8.3	62.5 ↑	1,470 ± 151 ↑	58 ± 6.3 ↓
GRA12D	ND	ND	ND	825 ± 81	92 ± 7.2
Δ*gra12D*	16 ± 1.5	0.0	100 ↑	688 ± 96	45 ± 4.6 ↓

a The percent survival, cyst burden, and reactivation rate are shown with different inocula (e.g., 2 × 10^6^). ND, not done. The arrows denote significant increase (upward pointing arrow) or decrease (downward pointing arrow).

The GRA12 gene family has expanded to five genes in *Toxoplasma*, compared to the related *Eimeria* that possesses only two GRA12 genes (41). Although the GRA12 gene family was initially discovered using ToxoDB data, identification of any new GRA protein is based on previously determined criteria outlined by Mercier and Cesbron-Delauw, which includes confirmation that the proposed GRA protein localizes in the dense granule organelles and also localizes within the PV (12). We showed that GRA12A, GRA12B, and GRA12D localized to the dense granule organelles, localized within the PV, colocalized with IVN markers GRA2 and GRA1, and were highly expressed in the tachyzoite and bradyzoite stages. GRA12C and GRA12B are neighboring genes on chromosome III. However, GRA12C is not detectably expressed during the tachyzoite or bradyzoite stage, and based on mRNA sequencing (mRNAseq) data, the GRA12C gene has peak expression during the merozoite and oocyst stages. Interestingly, the tandem duplication of GRA12C and GRA12B genes does not occur in *Neospora*, which has 81% genomic synteny to *Toxoplasma*, but within expanded gene sets, only 30% of overlap exists between the two species (53). This further suggests that GRA12C is specifically expressed in sexual stages in the feline definitive host gene, particularly since GRA12B and GRA12C are also neighboring duplicated genes in *Hammondia*, which shares the same definitive host as *Toxoplasma*.

Bright GRA12A, GRA12B, and GRA12D puncta were visible at the cyst periphery in 6-h differentiated cysts; similar localization has been seen with CST1, GRA1, GRA2, GRA4, GRA5, GRA6, GRA7, GRA9, GRA12 (30, 31), and succinylated wheat germ agglutinin (s-WGA)-stained molecule(s) (54). Together, this suggests an accumulation of GRA molecules at the cyst periphery early after differentiation occurs, prior to assembly of the cyst wall. Bradyzoites secrete vesicles into the cyst matrix, and similar vesicles are also observed near and within the cyst wall (34, 36). Bright GRA12A, GRA12B, and GRA12D puncta observed early after differentiation may represent cyst wall cargo-filled vesicles secreted by bradyzoites that are transported to the cyst periphery for building the cyst wall. How wall cargo is transported and incorporated into the cyst wall structure still remains to be determined.

Proteomic analysis of the composition of the cyst wall identified at least 22 GRA proteins, which included GRA12 (32). In this proteomic study, GRA12B was identified just below the arbitrary cutoff to be located in the cyst wall, along with GRA37 and additional hypothetical proteins, which are likely to be cyst wall proteins (32). Previously, GRA12 was localized to the outer and inner layers of the cyst wall during cyst development (30). GRA12 strongly colocalized with GRA2 in the cyst wall and with GRA2 in distinct puncta within the cyst matrix during cyst development (30). However, after 10 days of cyst development *in vitro*, GRA12 was not localized in the cyst wall, suggesting that GRA12 plays a late role in maturation of the cyst wall and/or cyst matrix (30). Our data in this study revealed that GRA12A and GRA12B occupied both the outer and inner layers of the cyst wall, similar to CST1, GRA2, GRA5, GRA6, GRA7, GRA12 (30, 31), and s-WGA-stained molecule(s) (54). In contrast, GRA12D primarily occupied the inner layer of the cyst wall in matured cysts, similar to GRA1, GRA4, and GRA9 (30). The presence of these molecules at the cyst wall suggests a function at the host-parasite interface.

The IVN membranes, which connect the parasites to the PV, are present in both the acute PV and in the chronic cyst, and in this stage are called the intracyst network (ICN) (34). In the tachyzoite stage, GRA12A, GRA12B, and GRA12D are colocalized with GRA2 within the PV, which suggests that GRA12-related proteins traffic through the IVN and are associated with the IVN. In the cyst stage, GRA12A, GRA12B, and GRA12D are colocalized with GRA2 within the cyst wall or the cyst matrix. GRA12A possesses a transmembrane domain, located at amino acids 64 to 86, and it is likely that GRA12A associates with membranes through this domain. In contrast, and similar to GRA12, GRA12B and GRA12D do not possess a typical transmembrane domain. How these GRA12 proteins associate with the IVN membranes remains to be identified.

Tubules that resemble the IVN membranes have also been previously reported in the cyst wall (34). The cyst matrix, the compartment between the bradyzoites and the cyst wall, is hypothesized to be an important supportive structure to the cyst wall and to the survival of bradyzoites. In the absence of GRA2 expression, there is a significant reduction in cyst burden (26). Previously, GRA2 localization was tracked during cyst development and the initial organization of the cyst matrix was observed in 3-day-old *in vitro* cysts (30). Furthermore, genetic deletion of GRA2 resulted in the disorganization of the cyst matrix, as seen by disorganized staining patterns of GRA4 and GRA6 (30, 54). In the cyst matrix, GRA12A appeared as puncta in a pattern similar to those of GRA2 and GRA12, while GRA12B and GRA12D appeared in a continuous matrix pattern, similar to those of GRA4, GRA6, and GRA9 (30). The function of many IVN GRA proteins is hypothesized to be involved in acquisition of nutrients from the host and distribution of nutrients to the bradyzoites. GRA2 and GRA6, through lipid binding (55), form membrane tubules that are involved in heterophagic ingestion of host cell proteins (56), Rab-positive host cell vesicles (57), and Rab7-positive host lipid droplets (20). Acquisition of host cell cargo and digestion are also likely to be essential for chronic infection (58), and recently published evidence supports the hypothesis that encysted bradyzoites have the capability to import host cell proteins and cargo (59).

Genetic deletion of GRA12A and GRA12B strongly influenced cyst burden in chronic infection. Deletion of GRA12A reduced cyst burden by 59%, while GRA12B increased cyst burden by 60%. The reduction of cyst burden by Δ*gra12A* parasites could be because fewer parasites successfully enter into the brain or because cysts are less stable and are disrupted upon brain homogenization. Deletion of a gene that causes an increase in cyst burdens is rare but has been previously observed after deletion of ROP16 (Δ*rop16*) (50). The increase in Δ*gra12B* cyst burden could be due to the increased replication rate of this mutant. Despite the increase in cyst burdens at 3 weeks postinfection with Δ*gra12B* parasites, this increased cyst burden was not maintained to 6 weeks postinfection, and compared to the complemented strain, Δ*gra12B* cyst burdens were not efficiently maintained. Further experiments are necessary to determine whether this phenotype is due to cyst instability or to increased clearance by host immune responses. It is unlikely that this phenotype involves increased cyst reactivation because Δ*gra12B* cysts exhibit a defect in their ability to reactivate.

As well as Δ*gra12B* cysts, Δ*gra12A* and Δ*gra12D* cysts also exhibit a defect in their ability to reactivate *in vitro*. In addition, Δ*gra12A* cysts exhibited a second reactivation defect, which was the delayed reactivation of cysts. The new *in vitro* cyst reactivation assay we developed is the first quantitative method that permits the measurement of individual cyst reactivation events as PFU. Consequently, we anticipate that this assay should permit the future observation of individual *in vitro* cysts in the process of reactivation with the goal to uncover steps and mechanisms that underpin cyst reactivation. Additional experiments are still needed to confirm that the *in vitro* reactivation defects we observed in Δ*gra12A*, Δ*gra12B*, and Δ*gra12D* cysts are also observed during *in vivo* infection of the host, as well as to investigate whether the defect in the percentage of Δ*gra12A*, Δ*gra12B*, and Δ*gra12D* cysts that reactivated *in vitro* is due to a loss of bradyzoite viability, a failure of bradyzoites to dedifferentiate into tachyzoites, or a failure to disrupt the cyst wall, which the parasites must first transverse in order to infect new host cells and form PFU. The delayed reactivation phenotype of Δ*gra12A* cysts could be caused by a delay in the disruption of the cyst wall or perhaps by a failure of the cyst wall to signal bradyzoites that cysts are now in more favorable growth conditions. Collectively, our results support the hypothesis that while certain GRA12-related proteins associated with the intravacuolar membrane system such as GRA12 supports parasite virulence during acute infection, other membrane-associated GRA12-related proteins primarily support the development of cyst burdens, cyst persistence, and cyst reactivation during chronic infection.

## MATERIALS AND METHODS

### Culture conditions and strains.

Type II Prugniaud (Pru) parasites were maintained *in vitro* by serial passage of tachyzoites in human foreskin fibroblast (HFF) monolayers (ATCC SCRS-1041.1) cultured in Eagle’s modified essential medium (EMEM) (Lonza) containing 1% fetal bovine serum (FBS) (Life Technologies), 2 mM glutamine, 100 U/ml penicillin, and 100 μg/ml streptomycin at 36°C in 95% air and 5% CO_2_. HFF cells were maintained in EMEM, 10% FBS (HyClone), 2 mM glutamine, 100 U/ml penicillin, and 100 μg/ml streptomycin at 37°C in 95% air and 5% CO_2_. The PruΔ*ku80* strain (Table 2) was developed from a Pru parental strain that selectively expresses the green fluorescent protein (GFP) in the bradyzoite stage under the control of the lactate dehydrogenase gene 2 (LDH2) bradyzoite stage-specific promoter (38, 46, 47). Strains used in this study were developed using the Δ*ku80* knockout strain of the type II Pru strain as previously described (Table 2) (26, 38, 60).

**TABLE 2:** Genotypes of strains used or developed in this study

Strain	ToxoDB (TgME49 locus)	Parent	Reference
PruΔ*ku80*Δ*hxgprt*^*a*^		PruΔ*ku80*::*HXGPRT*	38
PruΔ*ku80*Δ*hxgprt*Δ*gra12A*::*HXGPRT*	TgME49_220890	PruΔ*ku80*::*HXGPRT*	This study
PruΔ*ku80*Δ*hxgprt*Δ*gra12A*::GRA12A^HAx3-FLAG^	TgME49_220890	PruΔ*ku80*Δ*hxgprt*Δ*gra12A*::*HXGPRT*	This study
PruΔ*ku80*Δ*hxgprt*Δ*gra12B*::*HXGPRT*	TgME49_275860	PruΔ*ku80*::*HXGPRT*	This study
PruΔ*ku80*Δ*hxgprt*Δ*gra12B*::GRA12B^HAx3-FLAG^	TgME49_275860	PruΔ*ku80*Δ*hxgprt*Δ*gra12B*::*HXGPRT*	This study
PruΔ*ku80*Δ*hxgprt*Δ*gra12C*::*HXGPRT*	TgME49_275850	PruΔ*ku80*::*HXGPRT*	This study
PruΔ*ku80*Δ*hxgprt*Δ*gra12C*::GRA12C^HAx3-FLAG^	TgME49_275850	PruΔ*ku80*Δ*hxgprt*Δ*gra12C*::*HXGPRT*	This study
PruΔ*ku80*Δ*hxgprt*Δ*gra12D*::*HXGPRT*	TgME49_308970	PruΔ*ku80*::*HXGPRT*	This study
PruΔ*ku80*Δ*hxgprt*Δ*gra12D*::GRA12D^HAx3-FLAG^	TgME49_308970	PruΔ*ku80*Δ*hxgprt*Δ*gra12D*::*HXGPRT*	This study

a This parental strain expresses GFP in bradyzoites under the lactate dehydrogenase gene 2 (LDH2) promoter.

### Identification of GRA12-related genes.

The *Toxoplasma* genome resource (www.toxodb.org) was used to identify genes similar to GRA12 (TgME49_288650) in all Coccidian species. The *Toxoplasma* GRA12-related genes include GRA12A (TgME49_220890), GRA12B (TgME49_275860), GRA12C (TgME49_275850), and GRA12D (TgME49_308970).

### Deletion of GRA12-related genes.

Targeted GRA gene of interest (GOI) deletions (Table 2) were developed using the PruΔ*ku80* strain as previously described (38, 50, 60) (see Table S1 in the supplemental material). Briefly, GRA gene locus knockout targeting plasmid was assembled in yeast shuttle vectors pRS416 using yeast recombinational cloning to fuse in order three distinct PCR products with 33-bp crossovers; a 5′ *GRA(GOI)* target gene flank, the *HXGPRT* selectable marker, and a 3′ *GRA(GOI)* target flank (see Fig. S3A in the supplemental material) (61). Knockout plasmids were engineered to delete at least 200 nucleotides of 5′ untranslated region (UTR) and the complete coding region of the *GRA(GOI)* gene locus as defined in the ToxoDB.org database (41). All oligonucleotide primers used to construct knockout targeting plasmids and the ToxoDB nucleotide definition of GRA gene locus deletion are listed in Table S1. Targeting plasmids were validated by DNA sequencing, and the plasmids were linearized at restriction sites inserted at the 5′ end of the 5′-targeting flank (Fig. S3A). Linearized targeting plasmids were transfected by electroporation into tachyzoites of the PruΔ*ku80* strain. *GRA(GOI)* knockouts were selected in 50 μg/ml mycophenolic acid and 50 μg/ml xanthine. Drug-selected strains were cloned by limiting dilution 30 days after transfection. *GRA(GOI)* knockouts were validated by genotype analysis using a PCR strategy (shown in Fig. S3A) to measure the following: (i) in PCR 1, targeted deletion of the coding region of the targeted gene (DF and DR primers); (ii) in PCR 3, correct targeted 5′ integration (CXF and 5'DHFRCXR primers); and (iii) in PCR 4, correct targeted 3′ integration (3'DHFRCXF and CXR primers) using knockout validation primers shown in Table S2.

10.1128/mSphere.00182-21.9 TABLE S1 Primers used to construct GRA knockouts and complementation. Download Table S1, PDF file, 0.07 MB. Copyright © 2021 Guevara et al. 2021 Guevara et al. https://creativecommons.org/licenses/by/4.0/ This content is distributed under the terms of the Creative Commons Attribution 4.0 International license.

10.1128/mSphere.00182-21.10 TABLE S2 Primers used to validate GRA knockouts and complementation. Download Table S2, PDF file, 0.05 MB. Copyright © 2021 Guevara et al. 2021 Guevara et al. https://creativecommons.org/licenses/by/4.0/ This content is distributed under the terms of the Creative Commons Attribution 4.0 International license.

### Complementation of Δ*gra12*-related gene knockout mutants.

Complementation plasmids were designed to complement Δ*gra12A*, Δ*gra12B*, Δ*gra12C*, or Δ*gra12D* through targeted chromosomal integration and expression of wild-type GRA12A, GRA12B, GRA12C, or GRA12D at the endogenous locus as previously described (Fig. S3B) (38, 50). Complementation plasmids were developed in the pRS416 yeast shuttle vectors using yeast recombination to fuse, in order, a 5′ UTR target flank, the complementing gene of interest, and the 3′ UTR target flank (Fig. S3B). Oligonucleotide DNA primers (Table S1) were used to generate the complementing genes, synthesized as one PCR product. Following plasmid assembly by yeast recombinational cloning, targeting plasmids were validated by DNA sequencing. Prior to transfection, plasmids were linearized via the unique restriction site at the 5′ end. Parasites were cultured for 2 days in normal infection medium, and cultures were then switched to selection medium containing 30 mg/ml of 6-thioxanthine (6TX) and were cloned 30 days after transfection by limiting dilution. Accurate targeting of complementing genes into the endogenous locus was validated by genotype analysis using PCR assays (strategy shown in Fig. S3B) to measure the following: (i) in PCR 1, insertion of gene; (ii) in PCR 5, correctly targeted 5′ integration; and (iii) in PCR 6, correctly targeted 3′ integration of the complementing gene at the endogenous locus using oligonucleotide DNA validation primers (Table S2).

### Oligonucleotide primers and PCR.

*Toxoplasma* genomic DNA was purified from tachyzoites with the QIAMP blood DNA minikit (Qiagen) on a robotic Qiacube (Qiagen). PCR products were amplified from primers (Integrated DNA Technologies) using high-fidelity polymerases (Roche). Primers to validate knockout and complemented genes (Table S2) were as follows: PCR 1 (dF-dR), PCR 2 (CxR-ExF), PCR 5 (CxF-dR), and PCR 6 (dF-CxR) (Fig. S3). The positive control was PruΔ*ku80* DNA with primers, and the negative control was PruΔ*ku80* DNA with no primers (Fig. S3). The Δ control was the deleted Δ*gra* strain with primers (Fig. S3). The *Toxoplasma* genome resource (www.toxodb.org) was used to identify gene loci of interest and sequences to design oligonucleotide primers (Table S2).

### Immunofluorescence assay.

For visualization of dense granule proteins in the dense granule organelles, freshly lysed tachyzoites were incubated for 30 min on poly-l-lysine (Sigma)-coated circular micro cover glass (Electron Microscopy Sciences). Nonattached tachyzoites were washed away with Dulbecco’s phosphate-buffered saline (DPBS) supplemented with Ca^2+^ and Mg^2+^ (HyClone). Samples were fixed in 4% paraformaldehyde for 20 min, permeabilized with 0.1% Triton X-100 for 10 min, and blocked with 10% FBS for 20 min at room temperature. For visualization of dense granule proteins within the PV, HFFs were cultured on circular micro cover glass and were infected with tachyzoites for 24 h. Samples were fixed in 4% paraformaldehyde for 10 min, permeabilized with 0.01% saponin (Sigma) for 10 min, and blocked with 10% FBS for 20 min. All samples were incubated with a 1:500 dilution of primary rabbit monoclonal anti-HA tag antibody (Cell Signaling) to stain GRA12-related proteins or with a 1:1,000 dilution of primary mouse monoclonal anti-GRA1, anti-GRA2, anti-GRA5 antibody (Biotem, Apprieu, France). Preparations were washed three times with DPBS supplemented with Ca^2+^ and Mg^2+^ and incubated 1 h at room temperature (RT) with a 1:1,000 dilution of secondary goat anti-rabbit and goat anti-mouse IgG antibodies conjugated to Alexa Fluor 488 and Alexa Fluor 594, respectively. All samples were mounted in SlowfadeGold antifade with DAPI (4′,6′-diamidino-2-phenylindole) (Life Technologies) and imaged at 100× with a Nikon A1R SI confocal microscope (Nikon, Inc.). Tachyzoites or vacuoles were located using differential interference contrast (DIC) microscopy. Confocal images as raw .nd2 files were imported and minimally processed for brightness in Fiji (62).

### Cell fractionation and Western blotting.

Vacuole membranes were physically separated from soluble vacuole lumen molecules from PVs in infected HFFs using a previously described method (26, 63). Briefly, HFF monolayers in 150-cm^2^ flasks were infected with a multiplicity of infection (MOI) of 3 for 24 h. Infected HFFs were washed gently once with DPBS supplemented with Ca^2+^ and Mg^2+^ to remove residual medium. Cell monolayers were dislodged into cold phosphate-buffered saline (PBS) supplemented with Ca^2+^ and Mg^2+^ in the presence of protease inhibitor cocktail (Roche). Cells were recovered by low-speed centrifugation, and infected HFFs were mechanically disrupted by syringing through a 25-gauge needle to break HFFs and PVs and to release still intact parasites. Parasites and large host cell debris were removed by low-speed centrifugation at 2,500 × *g* for 10 min. The supernatant containing the soluble portion and the membranes of the PVs were then fractionated by ultracentrifugation at 100,000 × *g* for 1 h into the soluble high-speed supernatant (HSS) and the vacuole membrane high-speed pellet (HSP) fractions. After ultracentrifugation for 1 h at 100,000 × *g*, samples were concentrated using Amicon ultra 0.5 centrifugal filter device, equal fractions of the HSP and HSS were boiled in Tris-glycine sodium dodecyl sulfate (SDS) sample buffer (Novex) containing 2-β-mercapthoethanol and separated on 10% Tris-glycine wedgewell gels (Novex). Gels included lanes with a prestained protein ladder (PageRuler) and a biotinylated protein ladder (Cell Signaling). Proteins were transferred to nitrocellulose membrane using semidry via Trans-Blot Turbo Transfer System (Bio-Rad). Membranes were blocked for 1 h with 5% (wt/vol) nonfat dry milk in 1× Tris-buffered saline with 0.1% Tween 20 (TBS-T). Membranes were incubated overnight at 4°C with primary rabbit monoclonal anti-HA tag antibody (Cell Signaling) to stain GRA12-related proteins. After three washes in 1× TBS-T for 5 min, membranes were incubated for 1 h at RT with secondary antibody, anti-rabbit (1:2,000), and anti-biotin (1:1,000) conjugated to horseradish peroxidase. After three washes in 1× TBS-T for 5 min, signals were detected with LumiGLO chemiluminescence (Cell Signaling) and exposed to X-ray film.

### Intracellular replication rate assay.

Parasite growth rate was determined using methods described previously employing a direct parasite per vacuole scoring approach (38). Briefly, triplicate monolayers of HFFs were infected at an MOI of ∼0.2, and parasites were allowed to invade cells for 1 h. Monolayers were washed three times in DPBS to remove extracellular parasites. Tachyzoites per vacuole were scored in 50 randomly encountered vacuoles at 45 h postinfection in three independent experiments in triplicate. Significance was determined by a one-way analysis of variance (ANOVA) test, and *P* values were calculated by Student’s *t* test as follows: *, *P* < 0.05; NS, not significant.

### Mice.

Female 7- to 9-week-old C57BL/6 mice were obtained from Jackson Laboratories (Bar Harbor, ME) and were maintained under specific-pathogen-free conditions at the Center for Comparative Medicine and Research at the Geisel School of Medicine at Dartmouth.

### Acute virulence assay.

High viability type II parasites (tachyzoites) were isolated from 3-day infected HFF cultures as previously described (38, 61). Parasites were centrifuged at 900 × *g* for 7 min, washed, and counted in DPBS. Parasite viability was confirmed in PFU assays. Groups of four C57BL/6 mice were injected intraperitoneally (i.p.) with 2 × 10^6^ or 2 × 10^5^ tachyzoites. Mice were monitored for symptoms of infection for 30 days, and survival was evaluated using a Kaplan-Meier curve.

### Cyst differentiation *in vitro*.

Tachyzoites were differentiated *in vitro* into bradyzoites within cysts as previously described by Tobin and colleagues (44). The differentiation medium contained Roswell Park Memorial Institute medium (RPMI) without bicarbonate supplemented with 2.05 mM l-glutamine (HyClone), 20 mM HEPES (free acid) (IBI Scientific), 1% XL-glutamine (a long-lasting stable form of glutamine; VWR), 1% FBS, and 1% penicillin-streptomycin. The pH of differentiation medium was adjusted to 8.1 with sodium hydroxide and filter sterilized. HFF cells were cultured on circular micro cover glass until confluent (Electron Microscopy Sciences), and confluent monolayers were infected with type II Pru parasite at an MOI of ∼0.5. Infected HFF cells were washed 3 h after infection once in DPBS supplemented with Ca^2+^ and Mg^2+^ and incubated in differentiation medium for 6 h, 1 day, 2 days, 3 days, 7 days, or 10 days at 37°C in ambient air. The medium was changed on day 3 and day 7.

### Cyst immunofluorescence assay and cyst locating.

Infected HFF cells were fixed in 4% paraformaldehyde for 10 min, and the excess paraformaldehyde was quenched with 0.1 M glycine. Cells were simultaneously permeabilized and blocked in 3% FBS−0.2% Triton X-100 for 30 min at RT. Cysts generated from PruΔ*ku80*, Δ*gra12A*, Δ*gra12B*, and Δ*gra12D* strains were incubated with rhodamine-labeled Dolichos biflorus agglutinin (DBA) (dilution of 1:250) (Vector Laboratories) for 1 h at RT to evaluate the cyst wall. All other cysts were incubated with a 1:500 dilution of primary rabbit monoclonal anti-HA tag antibody (Cell Signaling) to stain GRA12-related proteins, and/or 1:1,000 dilution of primary mouse monoclonal anti-GRA2 antibody (43) (Biotem, Apprieu, France). Preparations were washed three times with DPBS and incubated 1 h at RT with a 1:1,000 dilution of secondary goat anti-rabbit (H+L) antibody conjugated to Alexa Fluor 647 (Thermofisher) and donkey anti-mouse IgG (H+L) antibodies conjugated to Alexa Fluor 568 (Cell Signaling) or a 1:250 dilution of rhodamine-labeled Dolichos biflorus agglutinin (Vector Laboratories). Samples were mounted in SlowfadeGold antifade with DAPI (Life Technologies) and imaged with a Nikon A1R SI confocal microscope (Nikon, Inc.) using an Apo TIRF 100× Oil DIC N20 objective. Cysts were randomly selected for analysis by locating cysts using DIC microscopy. Bradyzoite differentiation in cysts was confirmed by GFP^+^ bradyzoites. The focal place (from a z-stack) selected for quantification was from the middle of the cyst, where the cyst size is maximal. Raw .nd2 files of cyst images were imported into Fiji for processing. Images were minimally processed for brightness (image → adjust → color balance) in Fiji (62). The colocalization of GRA2 with GRA12A, GRA12B, or GRA12D was performed visually by analyzing each imaged cyst for spots of fluorescent overlap, and the percentage was calculated by images that showed colocalization divided by the total number of cysts imaged and multiplied by 100. The number (*n*) of cysts for each strain analyzed in each experiment is reported in each figure legend.

### Cyst wall definition and analysis.

The cyst wall region was identified and defined as previously described (30). The cyst wall outer region was identified by DBA or GRA2, while the inner region was determined by GFP, which identifies the bradyzoites within the cyst. The cyst wall region is defined by an outer and inner boundary determined by the first point less than 50% of maximum fluorescence intensity of DBA or GRA2 and GFP, respectively. The cyst wall region is marked by dotted vertical black lines, and the peak of DBA fluorescence is marked by a dotted magenta line. Next, we evaluated location of GRA12A, GRA12B, or GRA12D in comparison to the DBA- or GRA2-stained cyst wall using fluorescence intensity measured at the same time within the cyst. This cyst wall analysis was used to determine whether two proteins were observed in the same layer.

### Cyst total fluorescent intensity quantification assay.

Raw .nd2 files of cyst images were imported into Fiji to measure total fluorescence intensity at the cyst periphery and within the cyst interior as previously described (30). The cyst periphery was determined to be the cyst wall plus two layers, which were added to include proteins near the cyst wall but not yet incorporated into the cyst wall. Fluorescence for DBA and/or GFP, GRA12A, GRA12B, and GRA12D was measured in PruΔ*ku80*, Δ*gra12A*, Δ*gra12B*, Δ*gra12D*, Δ*gra12A*::GRA12A, Δ*gra12B*::GRA12B, and Δ*gra12D*::GRA12D strains. To measure background fluorescence, a circle was drawn using the freehand selection tool, and fluorescence was measured outside the cyst on three different sides. All data were imported into Excel to be further analyzed as previously described (30). DBA and GFP was measured for each cyst; however, the plotted DBA and GFP data for each time point is randomized from the cysts measured. All ratios were entered and graphed in Prism. A ratio of <1 means there is greater fluorescence intensity in the cyst interior compared to the cyst periphery, a ratio of 1 represents an equal fluorescence intensity at the cyst periphery to the cyst interior, and a ratio of >1 means there is greater fluorescence intensity at the cyst periphery than in the cyst interior. Significance was determined by a one-way ANOVA test, and *P* values were calculated with a Tukey test as follows: *, *P* < 0.05; ***, *P* < 0.001; ****, *P* < 0.0001; NS, not significant.

### Cyst fluorescence intensity profiles.

Raw .nd2 files of imaged cysts were imported into Fiji to measure fluorescence intensity parallel to the cyst wall as previously described (30). Images were cropped to isolate each cyst. A macro was written to generate a reliable mask of the cyst, slightly outside the wall using the DBA-rhodamine channel. The DBA-rhodamine channel was used to threshold the cyst, and holes were filled inside to obtain a continuous mask of the whole cyst. Successive layers were generated based on the original mask, growing or shrinking by dilate or erode morphological operations. Layers were generally one pixel thick. The fluorescence intensity of each region was measured for a selected fluorescence channel: DBA, GFP, GRA2, GRA12A, GRA12B, or GRA12D. The macro-generated layers within the cyst until the minimum area of the (shrinking) layer reached 1,000 pixels^2^. Layers were created by dilation to measure the fluorescence intensity outside the cyst, which provided the background fluorescence intensity. All data were imported into Excel to be further analyzed, as previously described (30). The calculated percentages of maximum fluorescence intensity and distance (in micrometers) values were imported and graphed in Prism.

### Quantification of colocalization.

Raw .nd2 files of imaged cysts (*n *= 7 cysts for each strain evaluated) were analyzed in Fiji with two programs, PSC Colocalization and Just Another Colocalisation Plugin (JACoP), to calculate the following: Pearson’s coefficient, Manders 1 (M1) and Manders 2 (M2) coefficients, Costes’ automatic threshold, Costes’ randomization, and Spearman’s rank correlation coefficient to analyze the level of overlap between GRA2 and GRA12A, GRA12B, or GRA12D as previously described (64, 65). Pearson’s correlation coefficient (PCC) measures the predictability of a relationship by analyzing pixel intensity from the population mean, so closer to 1 is a positive relationship, while closer to −1 is a negative relationship and close to 0 is predictive of no relationship. M1 and M2 provide information about how proteins correlate with one another. Costes’ automatic threshold identifies the optimal threshold value for correlation analysis. Costes’ randomization is a control as it randomizes the pixels and measures the PCC of randomized pixels to evaluate cooccurrence due to random chance. Spearman’s rank correlation coefficient (SRCC) is applied to pixel intensity ranks, instead of values as performed by PCC, and identifies relationships that may be correlated but perhaps not in a linear fashion. A value close to one indicates a strong correlation, while a value close to zero indicates no correlation.

### Cyst burden assays.

High viability type II tachyzoites were obtained as previously described (38, 61). Tachyzoites were centrifuged at 900 × *g* for 7 min, washed, and counted in DPBS. Groups of four C57BL/6 mice were injected intraperitoneally with 2 × 10^2^ parasites, and parasite viability was confirmed in a plaque assay. Mice were monitored for symptoms of infection for 3 or 6 weeks. Brains from mice infected with type II Pru strains were harvested postinfection and homogenized using a Dounce homogenizer in 2 ml sterile DPBS. Cyst counts were performed on a minimum of 10% of each brain. Since Pru strain background cysts can vary in size (38, 66), cysts were scored using dark field microscopy with an inverted fluorescence phase-contrast microscope (Olympus CKX41). The PruΔ*ku80* parent strain expresses GFP under the control of the bradyzoite stage-specific LDH2 promoter (38). GFP^+^ cysts were scored using a total magnification power of 150× that provided for the highest sensitivity of the detection of GFP^+^ bradyzoites within matured cysts. GFP^+^ cysts were then verified in bright field microscopy at 300× total magnification to also possess a translucent thick cyst wall surrounding the GFP^+^ bradyzoites (38, 61). The percentage of cysts maintained was calculated by the average number of cysts at 6 weeks divided by the average number of cysts at 3 weeks and multiplied by 100. Significance was determined by a one-way ANOVA test, and *P* values were calculated with a Tukey test as follows: **, *P* < 0.01; NS, not significant.

### Cyst reactivation assay.

Multiple confluent T-25 flasks of HFFs were infected with 2 × 10^2^ tachyzoites at 37°C and 5% CO_2_. After 3 h, all cultures were washed once with DPBS to remove noninvaded parasites, then one half of the cultures received 1% EMEM infection medium and were incubated undisturbed at 37°C with 5% CO_2_ in a PFU assay for 11 days. The other half of infected cultures received differentiation medium and were incubated for 3 days at 37°C without CO_2_. After 3 days of differentiation, the differentiated cultures were washed once with DPBS and received 1% EMEM infection medium, and cultures were incubated undisturbed at 37°C with 5% CO_2_ in a PFU assay for an additional 11, 13, 15, or 17 days. After the development of PFU, cultures were fixed and stained with methanol/Coomassie blue to reveal plaques. Images were taken with Lumix DMC-ZS60 digital photo camera at a magnification of 0.33. Plaque areas were measured in Fiji using a cropped section containing only plaques (rectangle selection + Ctrl-Shift-D). The cropped image was split into single channels (Image → Color → Split Channels, use Green). Using the green channel panel, process the image using a bandpass filter (Process → FFT → Bandpass Filter, use 100 pixels to filter large structures and 3 pixels to filter small structures). Then the image was thresholded (Ctrl-Shift-T) by an automatic method called MaxEntropy. Next, particles (size, 300 to 6000) were measured (Analyze → Analyze particles), and particles on the edge were excluded. At least 50 plaques were measured in each flask. Reactivation percent was calculated by calculating the average number of reactivated plaques divided by the average number of control plaques and multiplied by 100. Significance was determined by a one-way ANOVA test, and *P* values were calculated with a Tukey test as follows: *, *P* < 0.05; **, *P* < 0.01; ***, *P* < 0.001; ****, *P* < 0.0001; NS, not significant. The scale bar represents 100 pixels.

### Ethics statement.

All animal experiments were conducted in accordance with the recommendations in the *Guide to the Care and Use of Laboratory Animals* (67) and Association for the Assessment and Accreditation of Laboratory Animal Care (AAALAC) guidelines. Animals were housed in an AAALAC-certified facility, and animal protocols were approved by the Dartmouth College Committee on the Use and Care of Animals (Animal Welfare Assurance A3259-01, protocol 00002108).
