# Supplementary figures and images for: A Family of Toxoplasma gondii Genes Related to GRA12 Regulate Cyst Burdens and Cyst Reactivation
Source: mSphere. 2021 Apr 21;6(2):e00182-21. doi: 10.1128/mSphere.00182-21 (PMC8546695; doi:10.1128/mSphere.00182-21)

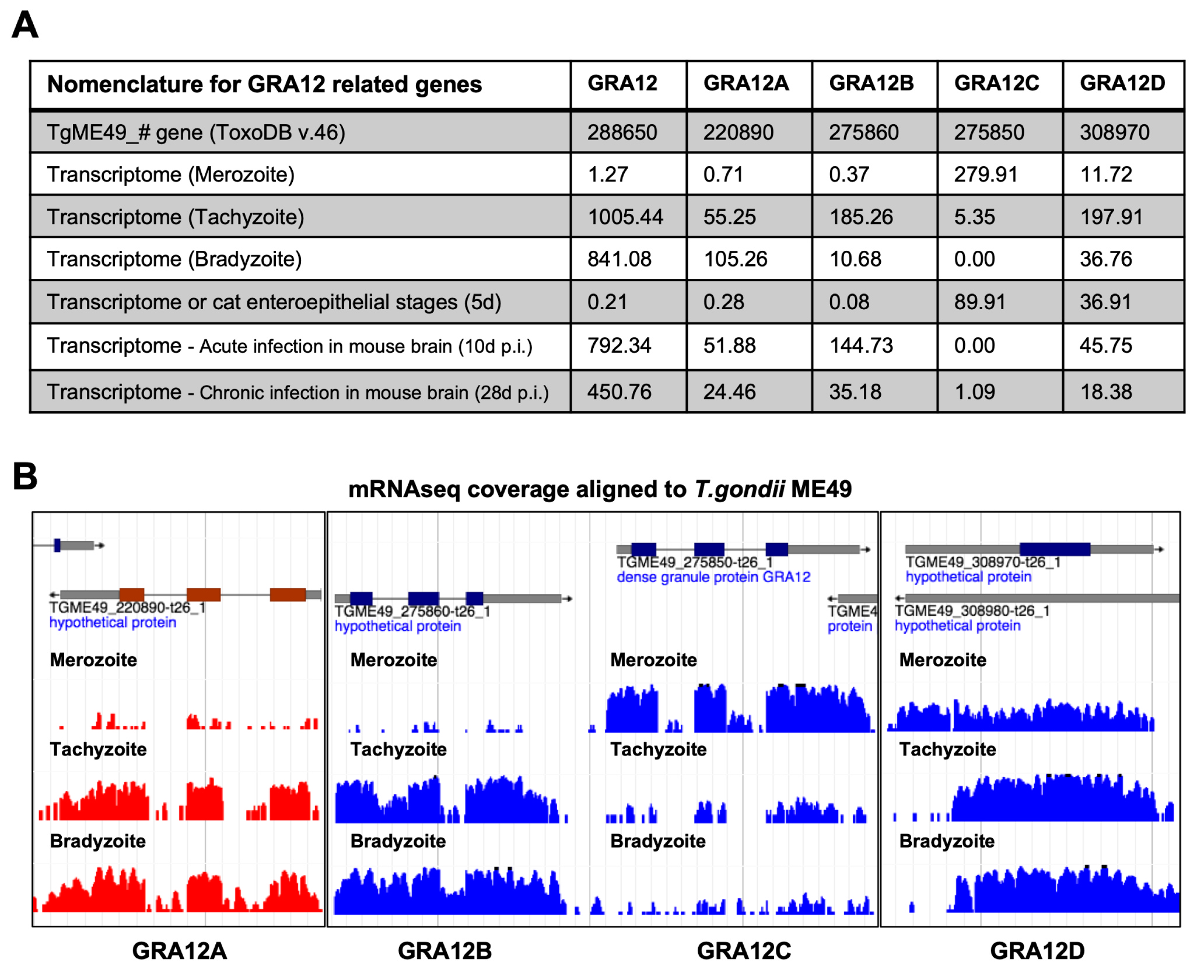

Supplement: FIG S2 [file msphere.00182-21-sf002.tif]

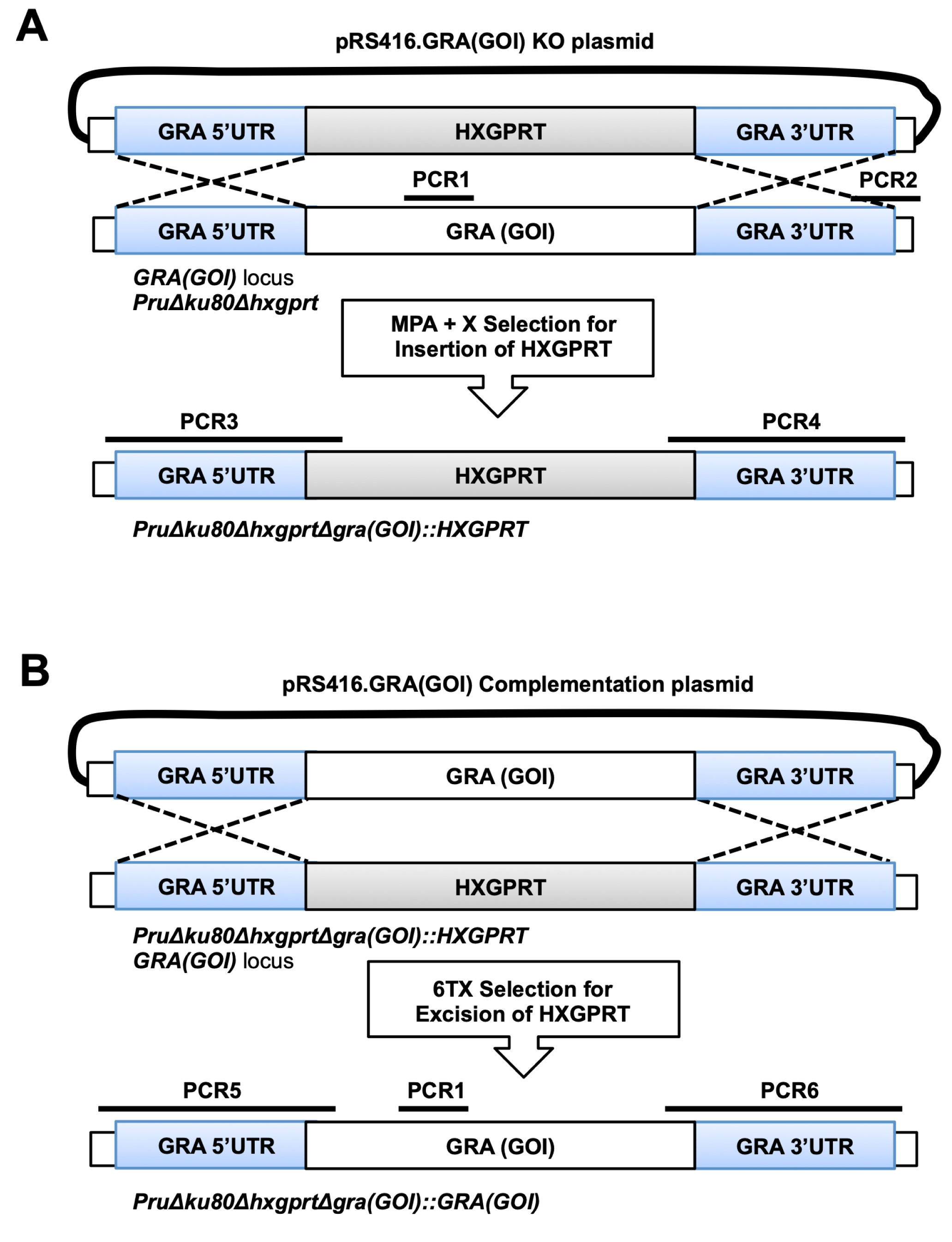

Supplement: FIG S3 [file msphere.00182-21-sf003.tif]

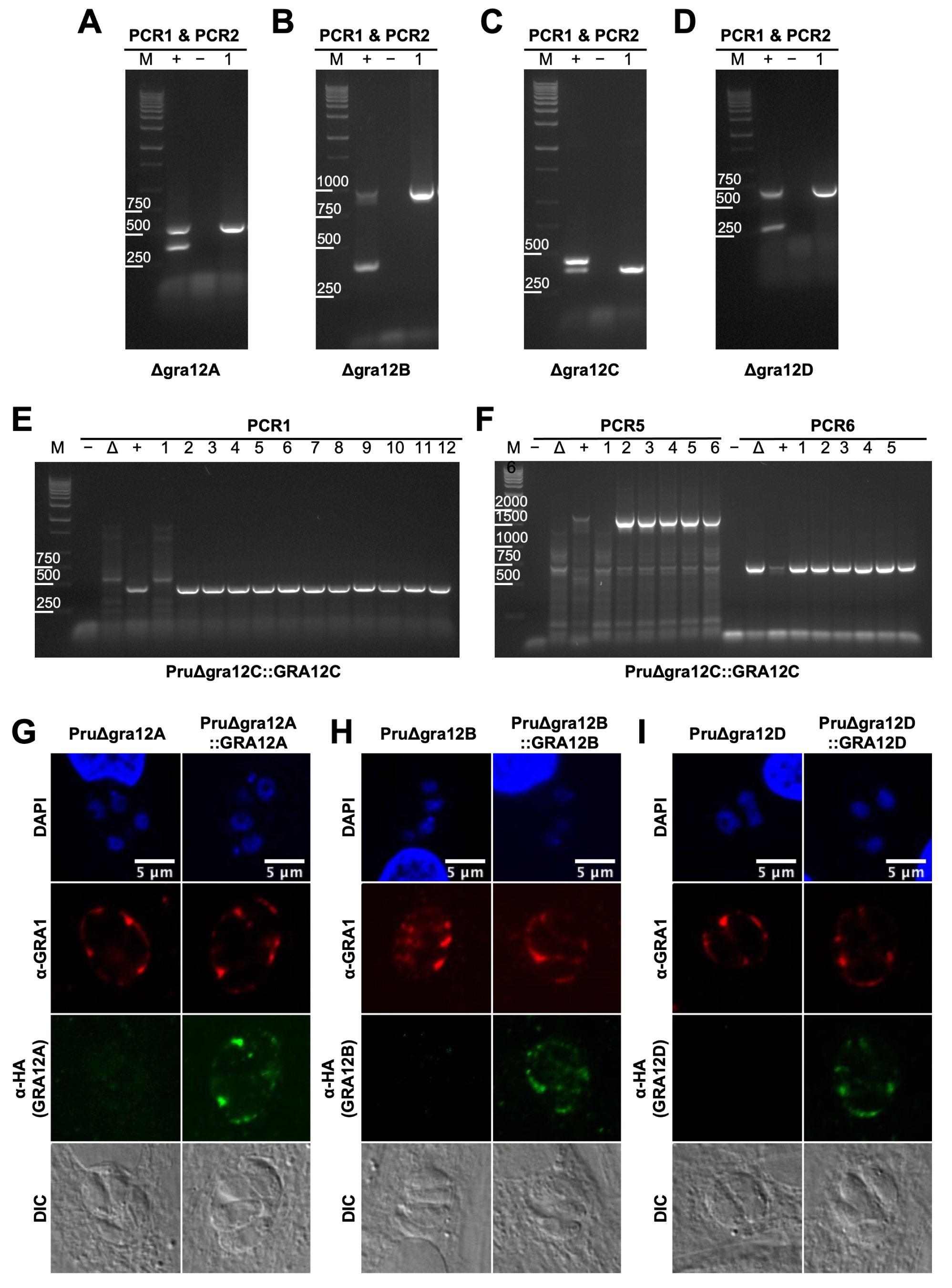

Supplement: FIG S4 [file msphere.00182-21-sf004.tif]

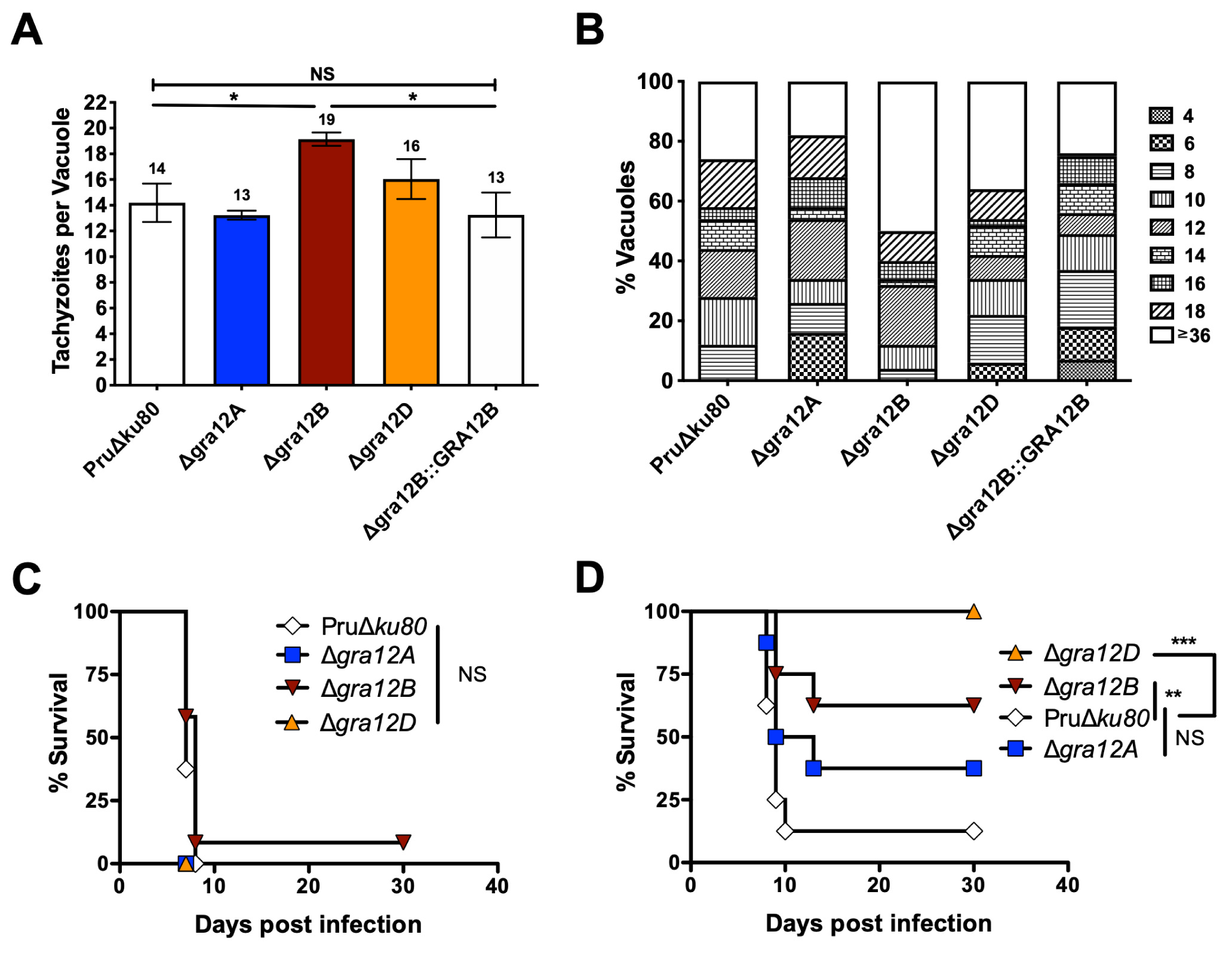

Supplement: FIG S5 [file msphere.00182-21-sf005.tif]

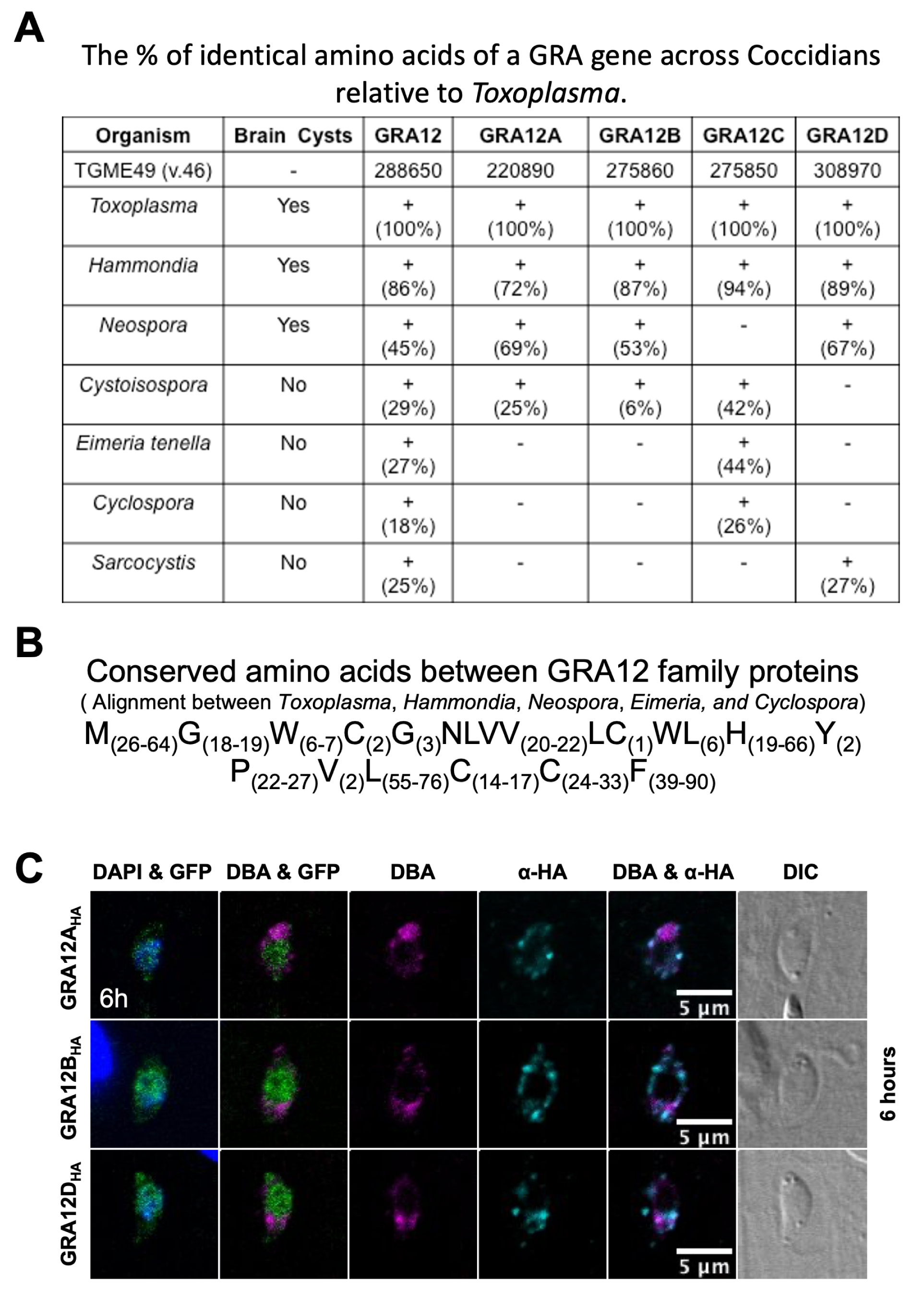

Supplement: FIG S6 [file msphere.00182-21-sf006.tif]

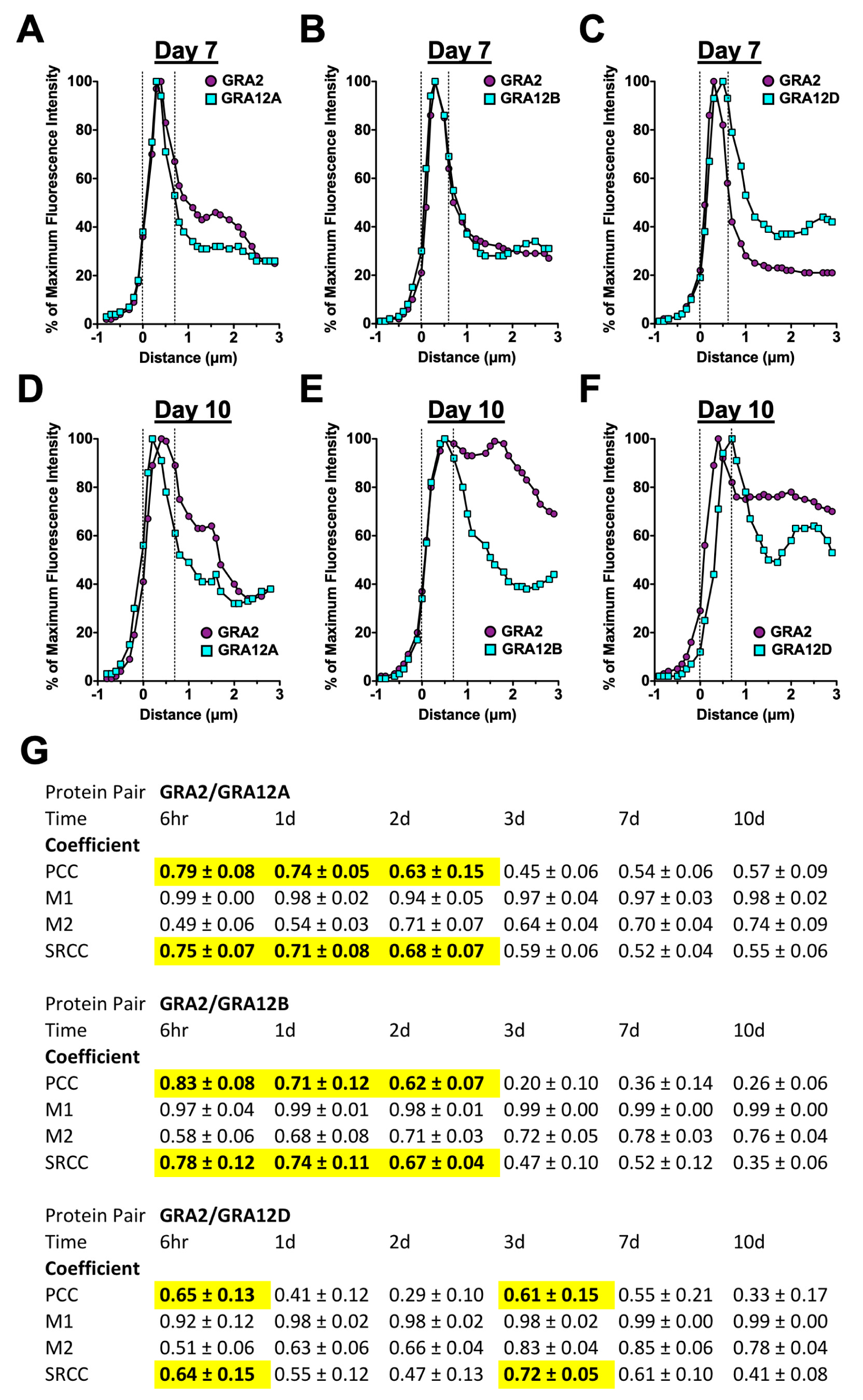

Supplement: FIG S7 [file msphere.00182-21-sf007.tif]
