# Supplementary material for: A Family of Toxoplasma gondii Genes Related to GRA12 Regulate Cyst Burdens and Cyst Reactivation
Source: mSphere. 2021 Apr 21;6(2):e00182-21. doi: 10.1128/mSphere.00182-21 (PMC8546695; doi:10.1128/mSphere.00182-21)
Supplement: TABLE S1 [file msphere.00182-21-st001.pdf]

Table S1. Primers used to construct *GRA* knockouts and complementations.

Primer	Primer Sequence	Primer Use	KO Construct	Corresponding TGME49 locus
GRA12AF1	TTGGGTAACGCCAGGGTTTTCCCAGTCACGACGTCTAGACACGATACGCTGTGGTGGGTC	Pru GRA12A KO 5' target FP	pRS416.GRA12A	TGME49_220890 chrII 37,902 to 41,699 (-)
GRA12AR1	GCGGGTTTGAATGCAAGGTTTCGTGCTGATCAAGTTTAAACGTCGCTAGAGCGTTTACCGTAACC	Pru GRA12A KO 5' target RP		
GRA12AF2	TTCTGGCAGGCTACAGTGACACCGCGGTGGAGGGTTTAAACGGAAGCCGGTGTTGGATTGGTC	Pru GRA12A KO 3' target FP		
GRA12AR2	GTGAGCGGATAACAATTTCACACAGGAAACAGCGCGGCCGCGCACACCACTTCCTTCTAAGTCCAC	Pru GRA12A KO 3' target RP		
GRA12BF1	TTGGGTAACGCCAGGGTTTTCCCAGTCACGACGACTAGTGGCTCAGCGTCACGTGAGAAG	Pru GRA12B KO 5' target FP	pRS416.GRA12B	TGME49_275860 chrIII 180,806 to 184,525 (+)
GRA12BR1	GCGGGTTTGAATGCAAGGTTTCGTGCTGATCAAGTTTAAACCTGCGAGCGCACTAAGCTCTG	Pru GRA12B KO 5' target RP		
GRA12BF2	TTCTGGCAGGCTACAGTGACACCGCGGTGGAGGGTTTAAACTCACAGATCCTGTAATACTGCGCTCC	Pru GRA12B KO 3' target FP		
GRA12BR2	GTGAGCGGATAACAATTTCACACAGGAAACAGCATTTAAATGTCCCACTGGTGAGCACGTTTC	Pru GRA12B KO 3' target RP		
GRA12CF1	TTGGGTAACGCCAGGGTTTTCCCAGTCACGACGACTAGTGAAACGTGCTCACCAGTGGGAC	Pru GRA12C KO 5' target FP	pRS416.GRA12C	TGME49_275850 chrIII 185,445 to 189,438 (+)
GRA12CR1	GCGGGTTTGAATGCAAGGTTTCGTGCTGATCAAGTTTAAACCATGCTTGCACCTCTTAGGCGAG	Pru GRA12C KO 5' target RP		
GRA12CF2	TTCTGGCAGGCTACAGTGACACCGCGGTGGAGGGTTTAAACCAGCATGTTCAGGTGGGTGAAGC	Pru GRA12C KO 3' target FP		
GRA12CR2	GTGAGCGGATAACAATTTCACACAGGAAACAGCATTTAAATCTTCCGTTTCGATGCCGGTCTC	Pru GRA12C KO 3' target RP		
GRA12DF1	TTGGGTAACGCCAGGGTTTTCCCAGTCACGACGATTTAAATGATCTTGCGGCCGGTACACAC	Pru GRA12D KO 5' target FP	pRS416.GRA12D	TGME49_308970 chrXI 408,907 to 412,526 (+)
GRA12DR1	GCGGGTTTGAATGCAAGGTTTCGTGCTGATCAAGTTTAAACCGGTCAACGGATGCGTGACC	Pru GRA12D KO 5' target RP		
GRA12DF2	TTCTGGCAGGCTACAGTGACACCGCGGTGGAGGGTTTAAACCATGACTATGTCATTCGCAGGTACCG	Pru GRA12D KO 3' target FP		
GRA12DR2	GTGAGCGGATAACAATTTCACACAGGAAACAGCGCGGCCGCCAGTCATCATGGGGAATTGTGATGGC	Pru GRA12D KO 3' target RP		
GRA12AF1	TTGGGTAACGCCAGGGTTTTCCCAGTCACGACGTCTAGACACGATACGCTGTGGTGGGTC	Pru GRA12A 5'target FP	pRS416.GRA12A	TGME49_ 220890 chrII 37,902 to 41,699 (-)
GRA12AR4	TCACTTGTCGTCGTCGTCCTTGTAGTCAGCGTAATCTGGAACATCGTATGGGTAAGCGTAATCTGG<br>AACATCGTATGGGTAAGCGTAATCTGGAACATCGTATGGGTAGAAGACCTTTTCTGGATTCAAAAT<br>GCCCT	Pru GRA12A coding+tag RP		
GRA12AF4	CGCTGACTACAAGGACGACGACGACAAGTGACGTGCATCGTAGATGGCAC	Pru GRA12A coding+tag FP		
GRA12AR2	GTGAGCGGATAACAATTTCACACAGGAAACAGCGCGGCCGCGCACACCACTTCCTTCTAAGTCCAC	Pru GRA12A 3'target RP		
GRA12BF1	TTGGGTAACGCCAGGGTTTTCCCAGTCACGACGACTAGTGGCTCAGCGTCACGTGAGAAG	Pru GRA12B 5'target FP	pRS416.GRA12B	TGME49_ 275860 chrIII 180,806 to 184,525 (+)
GRA12BR4	TTACTTGTCGTCGTCGTCCTTGTAGTCAGCGTAATCTGGAACATCGTATGGGTAAGCGTAATCTGGA<br>ACATCGTATGGGTAAGCGTAATCTGGAACATCGTATGGGTATGCTTTCCTAGCAGCTTCAACGTAGG	Pru GRA12B coding+tag RP		
GRA12BF4	CGCTGACTACAAGGACGACGACGACAAGTAAGGAGGCGCCTAGGGGTTG	Pru GRA12B coding+tag FP		
GRA12BR2	GTGAGCGGATAACAATTTCACACAGGAAACAGCATTTAAATGTCCCACTGGTGAGCACGTTTC	Pru GRA12B 3'target RP		

Table S1. Primers used to construct *GRA* knockouts and complementations.

Primer	Sequence	Primer Use	KO Construct	TGME49 Replacement Locus
**GRA12CF1**	*TTGGGTAACGCCAGGGTTTTCCCAGTCACGACGACTAGT***GAAACGTGCTCACCAGTGGGAC**	Pru GRA12C 5'target FP	pRS416.GRA12C	TGME49_ 275850 chrIII 185,445 to 189,438 (+)
**GRA12CR3**	**GAGAACTTGGGGCTGTCTGCC**	Pru GRA12C coding RP		
**GRA12CDF**	**GTGTTCGGTACATATGGCAGACAGC**	Pru GRA12C coding FP		
**GRA12CR4**	CTACTTGTCGTCGTCGTCCTTGTAGTCAGCGTAATCTGGAACATCGTATGGGTAAGCGTAATCTGG AACATCGTATGGGTAAGCGTAATCTGGAACATCGTATGGGTA**CTTCTTTTGTGAAGGTTTCATGAA CGTCG**	Pru GRA12C coding+tag RP		
**GRA12CF4**	CGCTGACTACAAGGACGACGACGACAAG**TAGGAAGCTGTGCACGCGG**	Pru GRA12C coding+tag FP		
**GRA12CR2**	*GTGAGCGGATAACAATTTCACACAGGAAACAGCATTTAAAT***CTTCCGTTTCGATGCCGGTCTC**	Pru GRA12C 3'target RP		
**GRA12DF1**	*TTGGGTAACGCCAGGGTTTTCCCAGTCACGACGATTTAAAT***GATCTTGCGGCCGGTACACAC**	Pru GRA12D 5'target FP	pRS416.GRA12D	TGME49_308970 chrXI 408,907 to 412,526 (+)
**GRA12DR4**	TCACTTGTCGTCGTCGTCCTTGTAGTCAGCGTAATCTGGAACATCGTATGGGTAAGCGTAATC TGGAACATCGTATGGGTAAGCGTAATCTGGAACATCGTATGGGTA**CAATTCACGGTACCTGC GAATGACATAG**	Pru GRA12D coding+tag RP		
**GRA12DF4**	CGCTGACTACAAGGACGACGACGACAAG**TGATTCGGTTTTGCAAGTCGAAAGTACC**	Pru GRA12D coding+tag FP		
**GRA12DR2**	*GTGAGCGGATAACAATTTCACACAGGAAACAGCGCGGCCGC***CAGTCATCATGGGGAATTGTGATGGC**	Pru GRA12D 3'target RP		

*Italicized nucleotides indicate regions of crossover in yeast recombination cloning, underlined nucleotides indicate restriction enzyme sites, and bold nucleotides indicate specific genomic priming target regions corresponding to type II ME49 in the *Toxoplasma gondii* database (ToxoDB version 30). FP indicates forward primer and RP indicates reverse primer. GRA12C replacement loci was pulled out in three parts.
