# Supplementary material for: A Family of Toxoplasma gondii Genes Related to GRA12 Regulate Cyst Burdens and Cyst Reactivation
Source: mSphere. 2021 Apr 21;6(2):e00182-21. doi: 10.1128/mSphere.00182-21 (PMC8546695; doi:10.1128/mSphere.00182-21)
Supplement: TABLE S2 [file msphere.00182-21-st002.pdf]

Table S2. Primers used to validate *GRA* knockouts and complementations.

Primer	Sequence	Primer use
5'DHFRCXR	ACTGCGAACAGCAGCAAGATCG	5' RP for validation of all GOI 5' flank integrations or for HXGPRT deletion validation
3'DHFRCXF	GTTGGCCTACGTGACTTGCTGATG	3' FP for validation of all GOI 3' flank integrations or for HXGPRT deletion validation
GRA12ACXF	CTGCAAGACCGCGAGCAAGC	FP for validation of GOI 5' flank integration
GRA12ADF	CACGACGGCACAGAAGCAGC	FP for GOI deletion validation
GRA12ADR	CATAGTCGTGAGCTTGGCCCTG	RP for GOI deletion validation
GRA12ACXR	CTGCTGGTGCCGCATGAGAC	RP for validation of GOI 3' flank integration
GRA12BCXF	CACGCAAGTCTCACCGCAGG	
GRA12BDF	CAGAGGTCGGTCTTACGCATGG	
GRA12BDR	CAGCTTGTGCCACAAGTACCACC	
GRA12BCXR	CGAACAGGAACGTGAGGTTCTCC	
GRA12CCXF	GGCACTCCAGCAGAGAAAGCAC	
GRA12CDF	GTGTTCGGTACATATGGCAGACAGC	
GRA12CDR	GTCTATGTGCATGGATGTCAGTGCG	
GRA12CCXR	CAGCCTCTGCTCTTGCTGCG	
GRA12DCXF	CGGTACCGACGAATCCACAGC	
GRA12DDF	CCGCAGGCGTACTGTTGGTAC	
GRA12DDR	CTGCACGTCGAGCCAGTCAC	
GRA12DCXR	GGACGCCGTGATAGTCCGTTC	

*FP indicates forward primer and RP indicates reverse primer. GRA(GOI)CXF is the FP for validation of GOI 5' flank integrations, GRA(GOI)DF is the FP for GOI deletion or integration validations, GRA(GOI)DR is the RP for GOI deletion or integration validations, GRA(GOI)CXR is the RP for validation of GOI 3' flank integrations.
